# The meiotic phosphatase GSP-2/PP1 promotes germline immortality and small RNA-mediated genome silencing

**DOI:** 10.1371/journal.pgen.1008004

**Published:** 2019-03-28

**Authors:** Katherine Kretovich Billmyre, Anna-Lisa Doebley, Maya Spichal, Bree Heestand, Tony Belicard, Aya Sato-Carlton, Stephane Flibotte, Matt Simon, Megan Gnazzo, Ahna Skop, Donald Moerman, Peter Mark Carlton, Peter Sarkies, Shawn Ahmed

**Affiliations:** 1 Department of Genetics, University of North Carolina, Chapel Hill, North Carolina, United States of America; 2 Department of Biology, University of North Carolina, Chapel Hill, North Carolina, United States of America; 3 Department of Genetics, University of Wisconsin-Madison, Madison, Wisconsin, United States of America; 4 Medical Research Council London Institute of Medical Sciences, London, United Kingdom; 5 Institute for Clinical Sciences, Imperial College London, London, United Kingdom; 6 Graduate school of Biostudies, Kyoto University, Kyoto, Japan; 7 Department of Zoology, University of British Columbia, Vancouver, British Columbia, Canada; Emory University, UNITED STATES

## Abstract

Germ cell immortality, or transgenerational maintenance of the germ line, could be promoted by mechanisms that could occur in either mitotic or meiotic germ cells. Here we report for the first time that the GSP-2 PP1/Glc7 phosphatase promotes germ cell immortality. Small RNA-induced genome silencing is known to promote germ cell immortality, and we identified a separation-of-function allele of *C*. *elegans gsp-2* that is compromised for germ cell immortality and is also defective for small RNA-induced genome silencing and meiotic but not mitotic chromosome segregation. Previous work has shown that GSP-2 is recruited to meiotic chromosomes by LAB-1, which also promoted germ cell immortality. At the generation of sterility, *gsp-2* and *lab-1* mutant adults displayed germline degeneration, univalents, histone methylation and histone phosphorylation defects in oocytes, phenotypes that mirror those observed in sterile small RNA-mediated genome silencing mutants. Our data suggest that a meiosis-specific function of GSP-2 ties small RNA-mediated silencing of the epigenome to germ cell immortality. We also show that transgenerational epigenomic silencing at hemizygous genetic elements requires the GSP-2 phosphatase, suggesting a functional link to small RNAs. Given that LAB-1 localizes to the interface between homologous chromosomes during pachytene, we hypothesize that small localized discontinuities at this interface could promote genomic silencing in a manner that depends on small RNAs and the GSP-2 phosphatase.

## Author summary

The germ line of an organism is considered immortal in its capacity to give rise to an unlimited number of future generations. To protect the integrity of the germ line, mechanisms act to suppress the accumulation of transgenerational damage to the genome or epigenome. Loss of germ cell immortality can result from mutations that disrupt small RNA-mediated genome silencing, which protects the germ line from foreign genetic elements such as transposons. Here we report for the first time that the *C*. *elegans* protein phosphatase GSP-2 that promotes core chromosome biology functions during meiosis is also required for germ cell immortality. Specifically, we identified a partial loss-of-function allele of *gsp-2* that exhibits defects in meiotic chromosome segregation and that is also dysfunctional for transgenerational small RNA-mediated genome silencing. Our results are consistent with a known role of *Drosophila* Protein Phosphatase 1 in heterochromatin silencing, and point to a meiotic phosphatase function that ensures germ cell immortality by promoting genomic silencing in response to small RNAs.

## Introduction

Animals, including humans, are comprised of two broad cell types: somatic cells and germ cells. Somatic cells consist of many diverse differentiated cell types, while germ cells are specialized to produce the next generation of offspring. An important difference between these two cell types is that somatic cells undergo aging phenomena while the germ line is effectively immortal and capable of creating new “young” offspring [1]. Understanding the basis of immortality in germ cells may provide insight into why organisms age.

In *C*. *elegans*, disruption of pathways that promote germ cell immortality results in initially fertile animals that become sterile after reproduction for a number of generations. Many such *mortal germline* (*mrt*) mutant strains are temperature-sensitive, becoming sterile at 25°C but remaining fertile indefinitely at 20°C [2]. Mutations that cause a Mrt phenotype have been reported in two distinct pathways: telomerase-mediated telomere maintenance [3,4] and small RNA-mediated nuclear silencing [5–9]. Mutations in the PIWI Argonaute protein cause immediate sterility in many species. However, disruption of the *C*. *elegans* Piwi orthologue PRG-1, which interacts with thousands of piRNAs to promote silencing of some genes and many transposons in germ cells, results in temperature-sensitive reductions in fertility and a Mrt phenotype [6–12]. Multiple members of a nuclear RNA interference (RNAi) pathway that promote the inheritance of transgene silencing also promote germ cell immortality and likely function downstream of PRG-1/Piwi and piRNAs [10,13]. One nuclear RNAi defective mutant, *nrde-2*, a number of heritable RNAi mutants, including *hrde-1*, and two RNAi defective mutants, *rsd-2* and *rsd-6*, only become sterile after growth for multiple generations at the restrictive temperature of 25°C [10,12–16]. The reason for this temperature-sensitivity is not clear. These ‘small RNA-mediated genome silencing’ mutants fail to repress deleterious genomic loci as a consequence of deficiency for small RNA-mediated memory of ‘self’ vs ‘non-self’ segments of the genome [13,17,18]. The transgenerational fertility defects of such mutants could reflect a progressive desilencing of heterochromatin, which is modulated by histone modifications that occur in response to small RNAs, such as H3K4 demethylation and H3K9me2/3 [15,19].

The SPR-5 histone 3 lysine 4 demethylase promotes genomic silencing in the context of H3K9 methylation and represses transgenerational increases in sterility [20]. Deficiency for *spr-5* also compromises germ cell immortality in a temperature-sensitive manner [21], similar to genome silencing mutants that are deficient for RNAi or RNAi inheritance [10,12–16]. However, thorough genetic screens for defects in RNAi inheritance failed to recover mutations in *spr-5* [16], and a direct test confirmed that deficiency for *spr-5* does not compromise RNAi inheritance [13]. It is therefore not clear if the role of SPR-5 and small RNA-mediated genome silencing proteins in maintenance of germ cell immortality is a consequence of deficiency for the same genomic silencing pathway. If this is the case, it is possible that deficiency for *spr-5* leads to the upregulation of a compensatory RNAi inheritance mechanism that masks an overt role for SPR-5 in RNAi inheritance.

Pioneering studies in *Neurospora* demonstrated that unsuccessful pairing of whole chromosomes during meiotic prophase, as well as discrete ‘unpaired’ chromosomal regions within paired meiotic homologs, can trigger small RNA-mediated genome silencing [22]. Multigenerational transmission of hemizygous transgenes in *C*. *elegans*, which results in an ‘unpaired’ ~10 kb genomic segment within paired homologous chromosomes during meiosis, leads to transgene silencing in a manner that depends on small RNAs and the PRG-1/Piwi Argonaute protein [23]. Therefore, a conserved small RNA mechanism operates during meiosis to promote genomic silencing when either large (chromosome scale) or small (transgene scale) segments of the genome are not properly paired.

A central function of Piwi/piRNA-mediated genomic silencing is to protect the genome from foreign genetic elements like transposons and viruses [11]. Horizontal transfer of a transposon into the genome of a naïve species will result in a burst of transposition events that ends when the host mounts a small RNA-mediated genomic silencing response against the transposon. In this context, *de novo* transposon insertions that represent a threat to genomic integrity would create small ‘unpaired’ hemizygous discontinuities within paired homologous chromosomes during meiosis. The discrete ‘unpaired’ meiotic chromosome aberrations created by *de novo* transposon insertions are structurally analogous to hemizygous transgenes, which are the targets of a multigenerational small RNA-induced genome silencing process [23]. Small ‘unpaired’ meiotic discontinuities created by *de novo* transposon insertions are therefore likely to be important for shaping genomic and epigenomic evolution.

*C*. *elegans* chromosomes do not have a discrete centromere to maintain cohesion between chromosomes during meiosis. Therefore they utilize two domains, separated by a crossover, called the long and the short arms. These arms separate at distinct stages of meiosis to prevent premature separation, with the short arms separating in Meiosis I and the long arms separating in Meiosis II. The regulation of cohesion occurs through localization of GSP-2 to the long arms of meiotic chromosomes through binding to LAB-1, where it antagonizes AIR-2 (Aurora-B kinase) activity [24–26]. In addition, LAB-1 is also present on mitotic chromosomes where it likely antagonizes AIR-2 activity [27]. In *C*. *elegans*, LAB-1 and GSP-2 fulfills the roles played by Shugoshin and Protein Phosphatase 2A in many other organisms, by protecting meiotic chromosome cohesion on the long arms in Meiosis I [27–29]. Once recruited by LAB-1, GSP-2 keeps REC-8, a meiosis-specific cohesin subunit, dephosphorylated to protect it from premature degradation and chromatid separation [26,27]. Additionally, recent work has shown that HTP-1/2, HORMA-domain proteins are responsible for LAB-1 chromosomal recruitment and therefore GSP-2 phosphatase activity [30].

Here we report the identification of a hypomorphic allele of *gsp-2*, a PP1/Glc7 phosphatase, which fails to maintain germline immortality at 25°C. GSP-2 is one of four PP1 catalytic subunits in *C*. *elegans* [31,32]. PP1 phosphatase has roles in many cellular processes including mitosis, meiosis, apoptosis and protein synthesis [33]. Previously, GSP-2 has been shown to promote meiotic chromosome cohesion by restricting the activity of the Aurora B kinase ortholog AIR-2 to the short arms of *C*. *elegans* chromosomes during Meiosis I [26,27]. Here, we demonstrate that GSP-2 is likely to act during meiosis to promote germline immortality via a small RNA-mediated genome silencing pathway.

## Results

### Identification of GSP-2 as a temperature-sensitive *mrt* mutant

**Fig 1:**
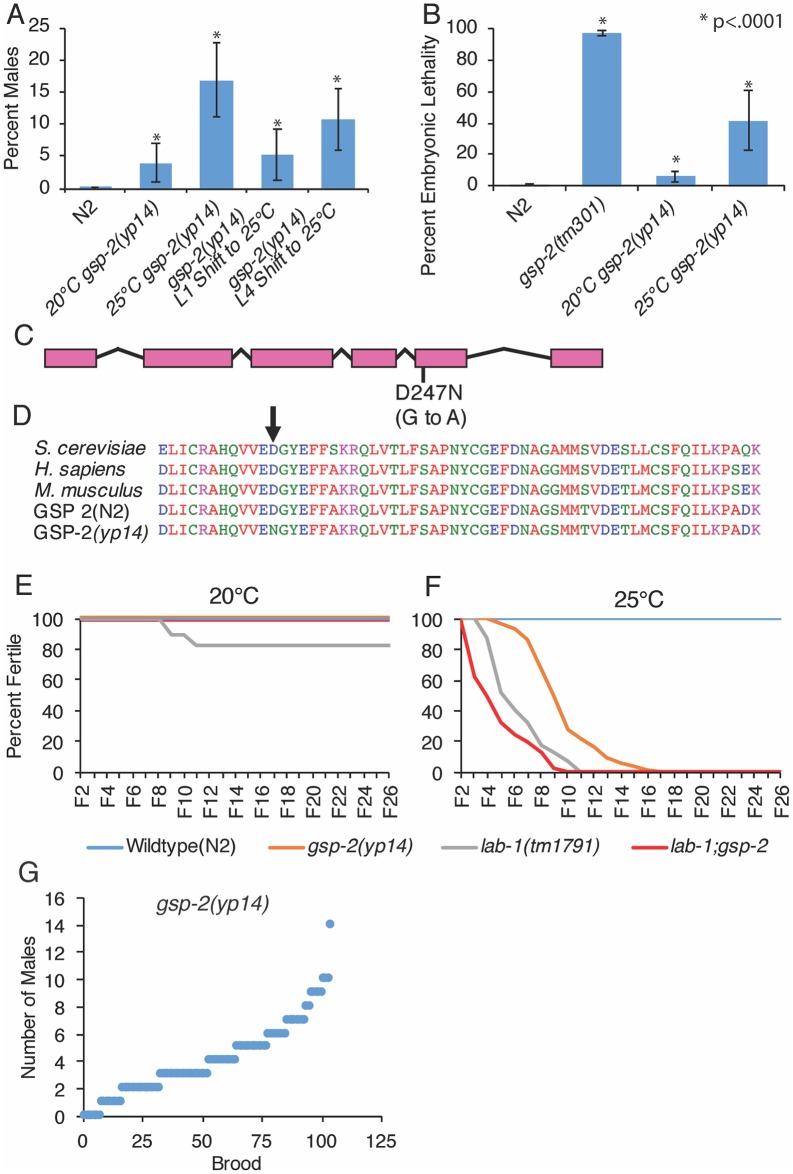
A hypomorphic mutation in *gsp-2* results in transgenerational sterility phenotype. **(A)** Incidence of males in *gsp-2(yp14)* was 3.9% at 20°C and increased to 16.8% at 25°C. When L1 animals were shifted to 25°C we saw a similar increase in males (5%, N = 42) to animals grown at 20°C and when L4 animals were shifted incidence of males was 10.7% (N = 49) **(B)** Progeny of *gsp-2(yp14)* animals grown at 20°C or 25°C were 6% and 41% Embryonic Lethal, respectively, compared to 97% of *gsp-2(tm301)* progeny (N = 20). **(C-D)** *gsp-2(yp14)* was identified to have a G to A mutation in exon 5 by whole genome sequencing. This results in a D to N amino acid substitution in a well conserved region of GSP-2. **(E)** When passaged at 20°C for many generations N2, *gsp-2(yp14)*, *lab-1* and *lab-1; gsp-2(yp14)* did not exhibit a loss of transgenerational fertility. **(F)** *gsp-2(yp14)* and *lab-1* both exhibited loss of fertility at 25°C and were completely sterile by generation F17 and F11 respectively. A double mutant of *lab-1;gsp-2* went sterile slightly faster than the individual single mutants and were completely sterile by F10. (N≥40) **(G)** Analysis of incidence of males showed no jackpots of males at in *gsp-2(yp14)* animals. *P <.0001 by T-test. Error bars represent standard deviation.

In a screen for *mrt* mutants [2], one mutation that displayed a Temperature-sensitive defect in germ cell immortality, *yp14*, was tightly linked to an X chromosome segregation defect manifesting as a High Incidence of Males (Him) phenotype, such that 3.9% of *yp14* self-progeny were XO males, which was significantly greater than the 0.05% male self-progeny phenotype observed in wildtype animals at 20°C (Fig 1A, p <.0001). The *yp14* mutation was mapped to Chromosome *III*, and whole genome sequencing revealed missense mutations in 6 genes within the *yp14* interval (S1A and S1B Fig). Three-factor mapping of the *yp14* Him and Mrt phenotypes suggested that *yp14* might correspond to the missense mutation in *gsp-2* (Fig 1C and 1D) or to a mutation in the G-protein coupled receptor gene *srb-11* (S1A and S1B Fig).

To test whether the chromosome segregation defect of *yp14* was due to a mutation in *gsp-2*, we performed a non-complementation test with a deletion mutation in *gsp-2*, *tm301*. *yp14 / tm301* F1 heterozygous hermaphrodites gave rise to F2 male progeny at a frequency of 5.7% at 20°C, similar to the 3.8% male phenotype observed for *yp14* homozygotes (S1C Fig). Thus, *tm301* failed to complement *gsp-2(yp14)* for its Him phenotype. In contrast, neither *gsp-2(tm301) / +* nor *gsp-2(yp14) / +* control animals displayed a Him phenotype (S1C and S1F Fig).

Additionally, *gsp-2(tm301)* null mutants immediately exhibited high levels of embryonic lethality at 20°C with a few F3 embryos that survive until adulthood (Fig 1B), consistent with roles for PP1 in chromosome condensation and segregation during mitosis in several species [24,25,34]. High levels of embryonic lethality for F3 *gsp-2(tm301)* mutant embryos (97%), led to uniformly sterile uniformly sterile F3 adults that produced no F4 progeny [25] (Fig 1B). These very high levels of embryonic lethality contrast with the embryonic lethality observed for *gsp-2(yp14)* mutants, which was 6% at 20°C and 41.6% for F8 animals grown at 25°C (Fig 1B). Both the Emb and Him phenotypes were exacerbated at 25°C (Fig 1A and 1B), suggesting that *gsp-2(yp14)* has a chromosome segregation defect that may be mechanistically linked to its Mortal Germline phenotype (Fig 1A and 1E).

In *gsp-2(yp14)* mutants, the X chromosome non-disjunction defect was more pronounced at both temperatures than the embryonic lethality associated with non-disjunction of the five *C*. *elegans* autosomes (S1 Table). Mutations that cause chromosome non-disjunction during mitosis occasionally lead to loss of an X chromosome during germ cell development, which could result in the stochastic appearance of XX hermaphrodites with high numbers of XO male progeny [35]. However, jackpots of XO males did not occur when *yp14* mutant hermaphrodites were isolated as single L4 larvae at 20°C or as L1 or L4 larvae at 25°C (Fig 1G, S1D and S1E Fig), implying that *yp14* is a separation-of-function mutation that specifically compromises the meiotic chromosome segregation function of GSP-2, with little or no effect on mitotic chromosome segregation. It is formally possible that *gsp-2(yp14)* is deficient for a mitotic function of GSP-2 that is relevant to germ cell immortality that is either distinct from its role in mitotic chromosome segregation or so subtle that we could not detect it in our assays.

### LAB-1 and GSP-2 promote germline immortality at high temperature

At 20°C, *gsp-2(yp14)* mutants remained fertile indefinitely, but at 25°C they exhibited sterility between generations F5 and F17 (Fig 1E and 1F). Given that LAB-1 promotes cohesion of the long arms of meiotic chromosomes via the GSP-2 phosphatase, we asked if LAB-1 is relevant to germ cell immortality by first outcrossing a *lab-1* deletion with wildtype and re-isolating *lab-1* homozygotes in an effort to eliminate epigenetic defects that could have accumulated in the parental *lab-1* strain. Outcrossed *lab-1* mutants displayed a Mortal Germline phenotype at 25°C (Fig 1E and 1F). We created *lab-1; gsp-2* double mutants, which remained fertile indefinitely when grown at 20°C but displayed a slightly accelerated number of generations to sterility at 25°C in comparison with *lab-1* mutants (Fig 1E and 1F). Together, these results suggest that a meiotic function of GSP-2 that is directed by LAB-1 promotes germ cell immortality. The small acceleration in the time to sterility in the double mutant animals suggests slight additivity between the mutations. Both the *gsp-2* and *lab-1* alleles are partial loss-of-function alleles that when combined could conceivably result in a stronger phenotype. Moreover, the weak Mortal Germline phenotype of *lab-1* single mutants at 20°C was suppressed by *gsp-2(yp14)* (Log Rank Test, p = .001). One possible explanation for this very slight rescue at the permissive temperature is the loss of *lab-1* alone results in GSP-2 being mis-localized and performing an ectopic function that is ablated when GSP-2 function is reduced. It is likely that this does not occur at 25°C because GSP-2 function is more severely compromised at the higher temperature.

### Small RNA-mediated genome silencing is disrupted in *gsp-2(yp14)*

**Fig 2:**
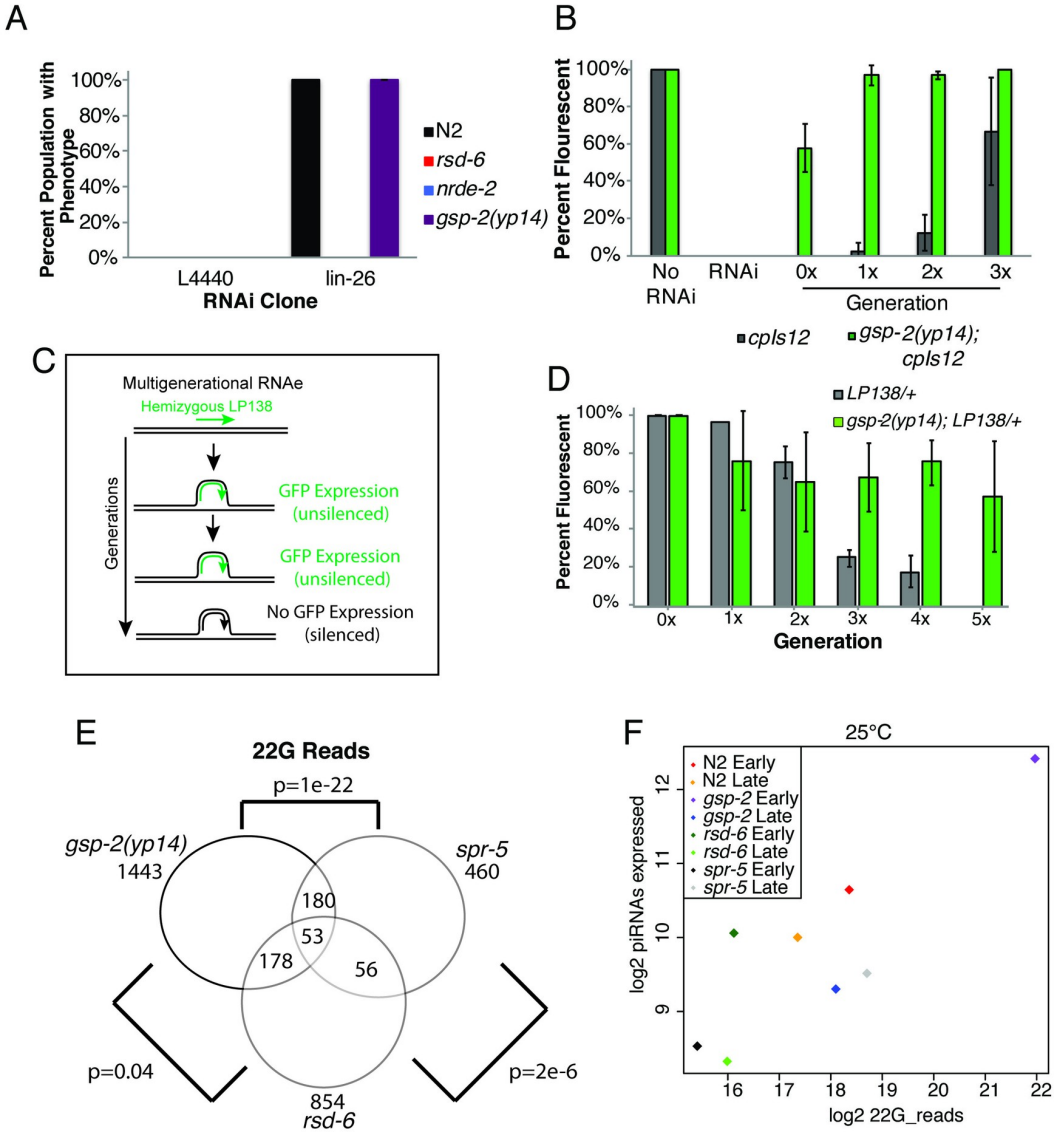
GSP-2 promotes multigenerational transgene silencing. **(A)** *gsp-2(yp14)* mutants do not exhibit single generation RNAi defects while *rsd-6* and *nrde-2* mutants are defective for single generation RNAi. **(B)** *cpIs12* treated with RNAi remains undetectable for multiple generations after RNAi treatment. However, in *gsp-2(yp14);cpIs12* animals treated with RNAi *cpIs12* only remains undetectable for one generation and by generation 3 exhibit close to wildtype levels of expression. **(C,D)** When LP138, a GFP transgene, is passaged as a heterozygote for multiple generations it is silenced in the germline. LP138 passaged as a heterozygote in a *gsp-2(yp14)* mutants results in only partial silencing over 5 generations suggesting defective heterozygous transgene silencing. **(E)** Comparison of small RNAs in *rsd-6*, *gsp-2* and *spr-5* mutants showing a great overlap in small RNA identity between *gsp-2* and *spr-5*. **(F)** Graph showing levels of piRNA expression in N2 controls, *rsd-6*, *gsp-2* and *spr-5* mutants at both early and late generations grown at 25°C.

Multiple genes that regulate small RNA-mediated epigenomic silencing promote germ cell immortality at high temperatures, like *gsp-2(yp14)* and *lab-1* [10,12,16]. Three small RNA-mediated epigenomic silencing genes that are required for germ cell immortality promote a specific form of transcriptional gene silencing termed nuclear RNA interference, *nrde-1*, *nrde-2* and *nrde-4* [10,12,36]. The response to a dsRNA trigger that targets *lin-26* is dependent on nuclear RNA interference [37]. Control wildtype and *gsp-2(yp14)* mutant animals displayed a completely penetrant Embryonic Lethality phenotype in response to *lin-26* dsRNA, whereas nuclear RNAi defective mutant *nrde-2* and the RNAi defective mutant *rsd-6* did not (Fig 2A), indicating that nuclear RNAi within a single generation is normal in the *gsp-2(yp14)* mutant.

Small RNAs can trigger RNAi inheritance [10,13], where silencing of a gene in response to siRNAs can be transmitted for multiple generations. Transgenerational RNAi inheritance can occur when endogenous genes are targeted by dsRNA triggers [38], but this can also happen when GFP reporter transgenes are targeted by small RNAs derived from *GFP* [13,17,18]. We tested the transgene *cpIs12 Pmex-5::GFP* and found that it was silenced in response to GFP siRNAs and that silencing of this transgene was inherited for up to 4 generations after removal from the dsRNA trigger (Fig 2B, Results summarized S6 Table). In contrast, GFP expression in *gsp-2(yp14)*; *cpIs12* was initially silenced but silencing was not inherited over multiple generations (Fig 2B), indicating that *gsp-2(yp14)* promotes RNAi inheritance.

Propagation of GFP or mCherry transgenes in the hemizygous state for multiple generations elicits a strong transgene silencing response, which is thought to be due to persistent yet small ‘unpaired’ discontinuities in the structure of paired meiotic homologous chromosomes at the site of the transgene [23]. We found that hemizygosity for the transgene *cpIs12* resulted in progressive transgene silencing in populations of animals over the course of several generations until *cpIs12* became fully silenced by generation 5 (Fig 2C and 2D). In contrast, when *cpIs12* was placed in a *gsp-2(yp14)* genetic background and propagated in a hemizygous state, we found that *cpIs12* was initially weakly silenced but that genomic silencing never became fully penetrant (Fig 2C and 2D). Together, the above data indicate that *gsp-2* promotes the silencing of unpaired hemizygous transgenes, which depends on small RNA-mediated genome silencing [23].

A central function of small RNA-mediated genomic silencing is to maintain silencing of repetitive elements and transposons in the germline, thereby protecting genomic integrity [15,19,39]. We previously reported that RNA expression of tandem repeat loci was upregulated in late-generation *rsd-2* and *rsd-6* mutants grown at 25°C [12]. Therefore, we asked if desilencing of tandem repeats occurred in *gsp-2(yp14)* mutants using RNA fluorescence *in situ* hybridization (FISH) to examine the expression of multiple repetitive elements. In wild-type controls grown at 25°C, we detected RNA from tandem repeat sequences using *CeRep59* sense and anti-sense probes in embryos but not in the adult germline or somatic cells, consistent with previous observations (S2 Fig) [12]. However, in late-generation *gsp-2(yp14)* and *rsd-6* mutants, robust expression of tandem repeats was observed throughout the soma and germline of adult animals, indicating that tandem repeats become desilenced in these strains (S2 Fig).

### Small RNA dysfunction in *gsp-2* mutants

Given that small RNA-mediated genome silencing is dysfunctional in *gsp-2(yp14)* mutants, we asked if small RNA populations were perturbed by preparing RNA from early- and late-generation wildtype, *gsp-2(yp14)*, *rsd-6* and *spr-5* mutants grown at either 20°C or 25°C. We examined *rsd-6* and *spr-5* mutants as they have known temperature sensitive germ cell immortality defects associated with loss genomic silencing as a consequence of small RNA or histone demethylation defects, respectively [12,21]. Small RNA libraries were prepared and subjected to high throughput sequencing, and we then examined levels of 22G RNAs that are 22 nucleotides in length beginning with a 5’ guanine, as 22G RNAs are the major effectors of genomic silencing in *C*. *elegans* [5,40]. 22G-RNAs in all late generation lines, normalized to total small RNA content showed a decrease relative to early generation N2 lines. The decrease was more pronounced in *gsp-2* and *rsd-6* mutants (p = 1.2e-7 and 4e-19, Wilcox paired test; S2 Table, S3 Fig) but not in *spr-5* where the decrease was not significantly different from the difference in N2 (p = 0.13). Analysis of the 22G-small RNA data revealed that *spr-5* and *rsd-6* share some genes with reduced levels of 22G RNAs with increasing generations, but there are other genes that show dissimilar behavior for each individual mutant. This suggests that *spr-5* may act both in conjunction with *rsd-6* and in a separate pathway to promote germline immortality. In contrast, 22G RNAs from *gsp-2(yp14)* showed strong similarities to those of *spr-5* mutants but showed little similarity to 22G RNA changes observed for *rsd-6* mutants, suggesting that *gsp-2(yp14)* and *spr-5* have similar effects on genome maintenance (S3 Fig). As a control, there is little coherent change in late-generation versus early generation N2 wildtype that overlaps with *gsp-2(yp14)* meaning that changes we see in *gsp-2(yp14)* are not due simply to passaging animals at 25°C (Fig 2E and 2F). As germ cell immortality is promoted in part by primary siRNAs termed piRNAs that interact with the Piwi Argonaute protein PRG-1 [8], we also examined piRNA populations, which are enriched for 21 nucleotide RNAs that begin with a 5’ uracil (21U RNAs) [6,7,9] and found that these were normal (Fig 2E and 2F). We also examined miRNAs, which have not previously been implicated in the Mortal Germline phenotype. Interestingly, miRNAs were significantly reduced in late generation *spr-5* and *gsp-2(yp14)* mutants (p = 1.2e-20 and p = 2.05e-25 respectively; S3 Table, S3 Fig), but not in *rsd-6* mutants. Since *spr-5* does not show global decrease in 22G-RNAs this is unlikely to be a secondary consequence of disturbance of the total small RNA pool. The relevance of this finding to the Mortal Germline phenotype awaits further investigation. Together these results indicate that *gsp-2(yp14)* and *spr-5* display common statistically significant changes in two classes of small RNAs, which implies that their genomic silencing defects may be more similar to one another than to those of *rsd-6* mutants.

### Small RNA silencing components and *gsp-2* promote germ cell immortality

**Fig 3:**
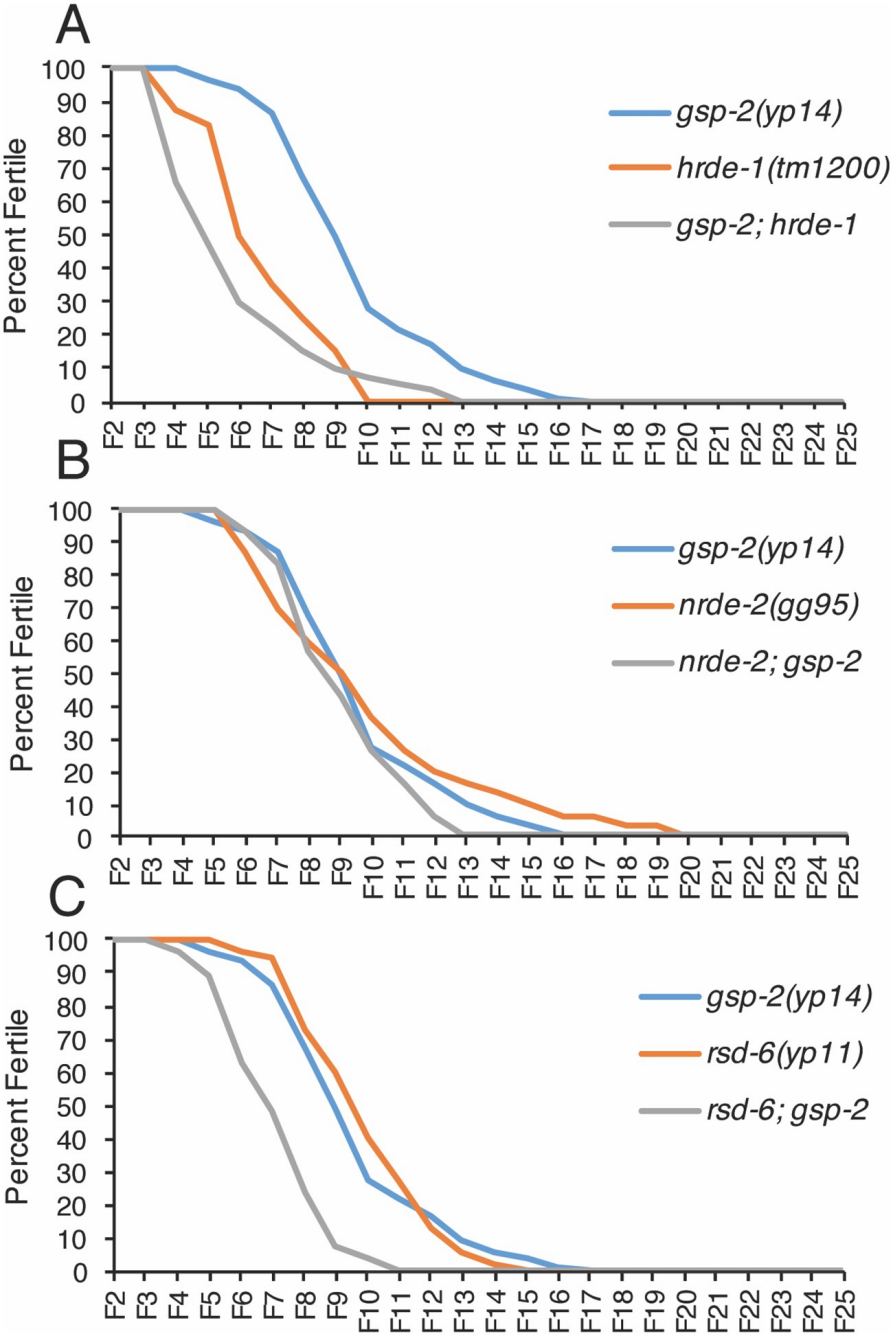
Temperature-sensitive small RNAi mutants exhibited similar times to sterility as *gsp-2(yp14)* at 25°C. Germline mortality assays all performed at 25°C **(A)** Both *gsp-2(yp14)* and *hrde-1* animals exhibit similar times to sterility while *gsp-2(yp14);hrde-1* double mutants display a slightly decreased time to sterility. p<.001 **(B)** *gsp-2(yp14)*, *nrde-2* and *rsd-6;gsp-2(yp14)* animals all go sterile in a similar number of generations. p = .06 **(C)** *gsp-2(yp14)* and *rsd-6* exhibit similar times to sterility while *gsp-2(yp14);rsd-6* double mutants become sterile at a slightly earlier generation. p<.001(N≥40). Significance was tested using a log rank test.

To study the relationship between *gsp-2(yp14)* and the small RNA genome silencing pathway, we created double mutants between *gsp-2(yp14)* and small RNA silencing mutants that display temperature-sensitive defects in germ cell immortality, *hrde-1*, *nrde-2* and *rsd-6*. Because *gsp-2(yp14)* is a hypomorphic allele, we predicted that single and double mutants would display a similar number of generations to sterility if it were functioning in the small RNA silencing pathway. For *gsp-2(yp14); hrde-1* and *rsd-6; gsp-2(yp14)*, we saw a modest decrease in the number of generations to sterility suggesting a slight additive effect (Fig 3A and 3C, Log Rank test: p <.0001). In contrast, *nrde-2; gsp-2(yp14)* double mutants did not differ from the single mutants (Fig 3B, Log Rank test: p = .06). Together, these results indicate that there is a weak additive effect on transgenerational lifespan when *gsp-2* is combined with *hrde-1* or *rsd-6*, but not when it is combined with *nrde-2*. The modest acceleration observed for some small RNA genomic silencing pathway and *gsp-2(yp14)* double mutants may be consistent with a single genome silencing pathway, as many single mutants in this pathway that display similar germline phenotypes at sterility also display a consistent, slightly accelerated sterility as double mutants. There are a number of explanations for this, including transmission of epigenetic defects from germ cells of the grandparents that created these double mutants, or shared but non-equivalent functions in terms of which segments of the genome each protein silences [15].

### Sterile *gsp-2* and *lab-1* mutants display germline defects similar to small RNA genome silencing mutants

**Fig 4:**
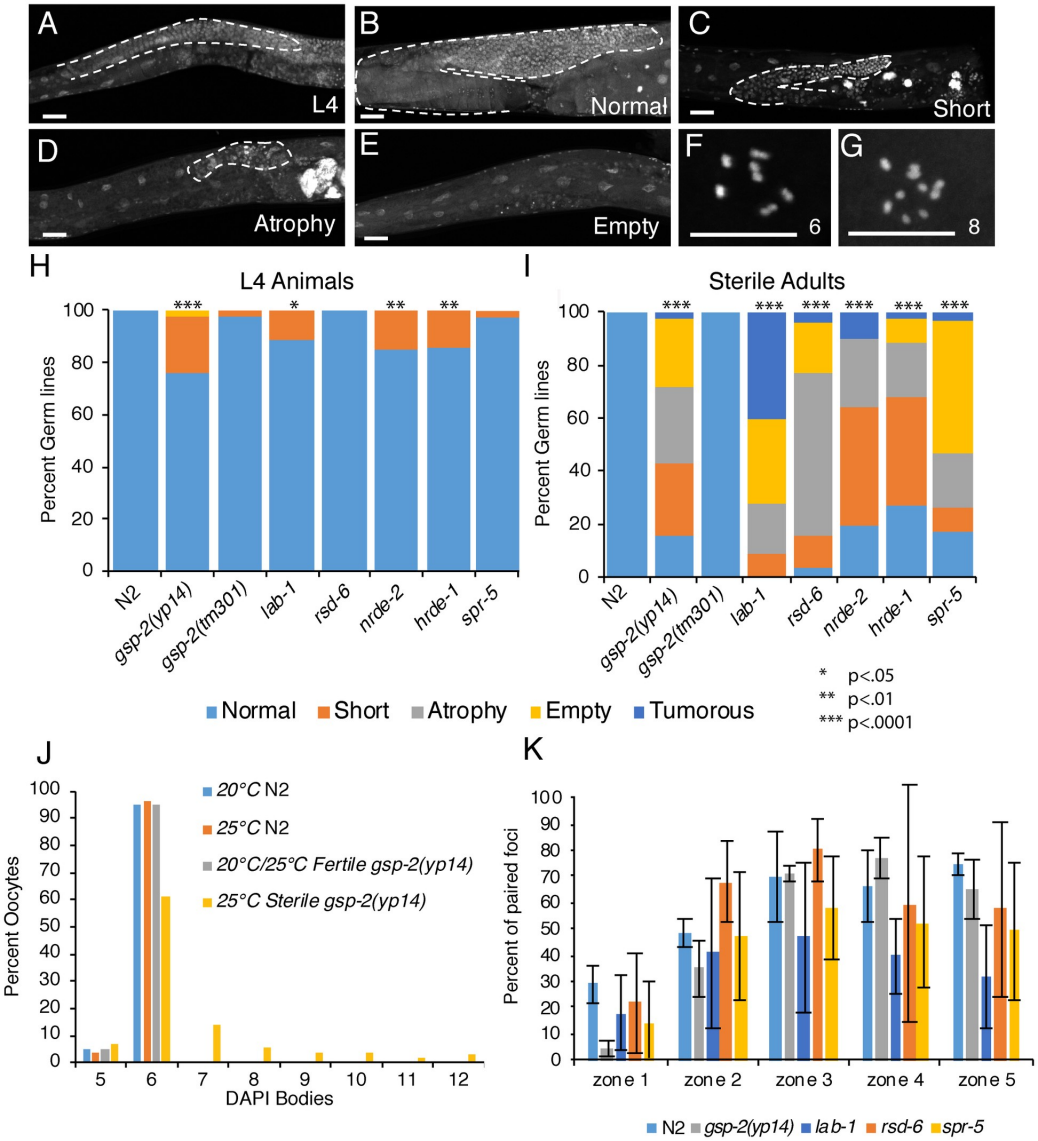
Germline defects occur in *gsp-2* and temperature-sensitive small RNA mutants at sterility. **(A-E)** Representative images of DAPI stained germlines passaged at 25°C until sterility. The timing of passage differed depending on the genotype as the time to sterility varies (See Fig 3). Germlines of either L4 (A) or adult control and sterile mutant animals were stained, and the germline size quantified as either normal (B), short (C), atrophied (D) or empty (E). **(F-G)** 6 DAPI bodies in control oocytes (F) and 8 DAPI bodies in *gsp-2(yp14)* animals (G). **(H-I)** Germlines from *gsp-2(yp14)*, *lab-1*, *rsd-6*, *nrde-2*, *hrde-1*, and *spr-5* mutants were examined and found to have mostly normal morphology at the L4 stage (H) but exhibited germline atrophy in adult animals (N≥98) (I). **(J)** In addition to germline atrophy, *gsp-2(yp14)* animals displayed greater than the wildtype number of 6 DAPI bodies in oocytes at the generation at sterility in 32% of oocytes (N≥100). **(K)** Quantification of HIM-8 staining showing the % of paired foci for each zone (See S2 Fig) along the germline for all indicated mutants. Error bars represent the standard deviation. P-values were obtained by using a student’s t-test for unpaired samples with unequal variance.

We previously reported that sterile, late-generation small RNA genome silencing mutants display a wide range of germline sizes, including many with few or no germ cells [12,41]. Therefore to investigate the cellular cause of transgenerational sterility in *gsp-2(yp14)* and *lab-1* mutants, we examined germline development in animals that became sterile after multiple generations. Most sterile generation L4 *gsp-2(yp14)* and *lab-1* mutant germlines were normal in size, though a small minority had a reduction in total germline length, resulting in a weak but significant difference in germline profile compared to wild-type (Fig 4A–4E and 4H, S5 Table, Results summarized S6 Table). Differentiating germ cell nuclei in spermatogenesis were observed for sterile generation L4 larvae for all strains (Fig 4A and 4H). However, the germlines of two-day-old sterile *gsp-2(yp14)* and *lab-1* mutant adults ranged in size from normal to a complete loss of germ cells (Fig 4B–4E and 4I), resulting in a significant difference when compared to wild-type controls (S5 Table p<1E-80). We studied small RNA genome silencing mutants and found that *rsd-6*, *hrde-1* or *nrde-2* mutant L4 larvae that were poised to become sterile displayed predominantly normal-sized germlines (Fig 4H). In contrast, sterile-generation *rsd-6*, *hrde-1* and *nrde-2* mutant adults had germline profiles that were similar to those of sterile *gsp-2(yp14)* mutant adults and markedly smaller than those of sterile generation L4 larvae (Fig 4I, S4 Table). *lab-1(tm1791)* displayed an increased frequency of germline tumors in comparison to other mutants, possibly due to a genetic modifier present in the *tm1791* mutant background.

Lastly, we tested if sterile *spr-5* mutants displayed similar germline phenotypes as those observed in small RNA mutants and *gsp-2(yp14)*. We found that sterile *spr-5* mutant adults displayed similar germline atrophy phenotypes, suggesting the resemblance to *gsp-2(yp14)* or *lab-1* mutants (Fig 4H and 4I). Our previous work showed that mutations in the cell death genes *ced-3* and *ced-4* partially rescued the empty and atrophy phenotypes observed for germlines of *rsd-2*, *rsd-6*, and *prg-1* small RNA genome silencing mutant adults [12,41], suggesting that apoptosis promotes germ cell atrophy as these animals develop from L4 larvae into adults.

To determine if acute loss of GSP-2 causes germline atrophy, we examined *gsp-2(tm301)* null mutants grown at 20°C and 25°C. *gsp-2(tm301)* homozygous F2 animals and their few surviving F3 progeny showed normal germlines, with no morphological defects in germline size or development for either L4 larvae or young adults, which significantly differed from the germline profiles of *gsp-2(yp-14)* animals (S4 and S5 Tables). Therefore, the late-generation sterility phenotype of *yp14* mutants is distinct from the fertility defects that occur in response to acute loss of GSP-2 in maternally depleted F3 deletion homozygotes.

### Pronounced meiotic defects occur in sterile *gsp-2(yp14)* mutants

Mature *C*. *elegans* oocytes typically contain 6 bivalents (pairs of homologous chromosomes held together by crossovers), which can be scored as 6 DAPI-stained bodies. Defects in meiotic pairing, cohesion, synapsis, and crossing over can lead to the presence of univalents, which are observed as greater than 6 DAPI bodies per oocyte. We previously observed that small RNA nuclear silencing *mrt* mutants *rsd-2* and *rsd-6* displayed increased levels of univalents at sterility, which were not observed in either wildtype or in fertile *rsd-2* or *rsd-6* mutant late-generation animals grown at 25°C [12]. We measured the presence of oocyte univalents in N2 wildtype control worms grown at 20°C and 25°C, which almost always contained 6 DAPI bodies representing the 6 paired chromosomes (5 bodies are occasionally scored when bivalents that overlap spatially cannot be distinguished). However, when *gsp-2(yp14)* worms were passaged at 25°C until sterility, only 60% of oocytes contained 6 paired chromosomes with the other 40% contained 7 to 12 DAPI bodies (Fig 4J, Results summarized S6 Table). This increase in oocyte univalents was not present in fertile *gsp-2(yp14)* worms, at 20°C or even for fertile late-generation 25°C *gsp-2(yp14)* adults that were close to sterility (Fig 4J). In contrast, we found no univalents in the null *gsp-2* allele *tm301*, either for F2 animals or for rare F3 escapers, consistent with previous observations [24,25].

LAB-1 has been previously implicated in the pairing of homologous chromosomes during meiosis [27]. To determine if homolog pairing is perturbed in *gsp-2(yp14)* mutants grown for two generations at 25°C, we examined the X chromosome pairing center protein HIM-8 in fertile 2 day old adults. When scored at pachytene only one spot was present in the majority of the nuclei suggesting pairing is occurring normally (Fig 4K, S4 Fig). In addition to *gsp-2(yp14)*, we examined HIM-8 foci in fertile *lab-1*, *rsd-6* and *spr-5* mutants grown at 25°C for two generations and found that *lab-1* mutants displayed decreased meiotic chromosome pairing consistent with previously reported data [27] but that pairing was relatively normal in the other mutants (Fig 4K, S4 Fig).

Given that LAB-1 and GSP-2 are known to promote meiotic chromosome cohesion, we tested the hypothesis that dysfunction of other factors that promote meiotic chromosome cohesion might be sufficient to elicit germline atrophy. Mutant strains with defects in cohesion, *smc-3(t2553)* and *coh-3(gk112); coh-4(tm1857)* double mutants [42–44] became sterile immediately and did not exhibit germline atrophy phenotypes observed in *gsp-2(yp14)* (S5 Fig, S4 Table). Therefore, the late-generation sterility phenotypes of *gsp-2(yp14)* and small RNA mutants are not due to acute loss of meiotic chromosome cohesion.

**Fig 5:**
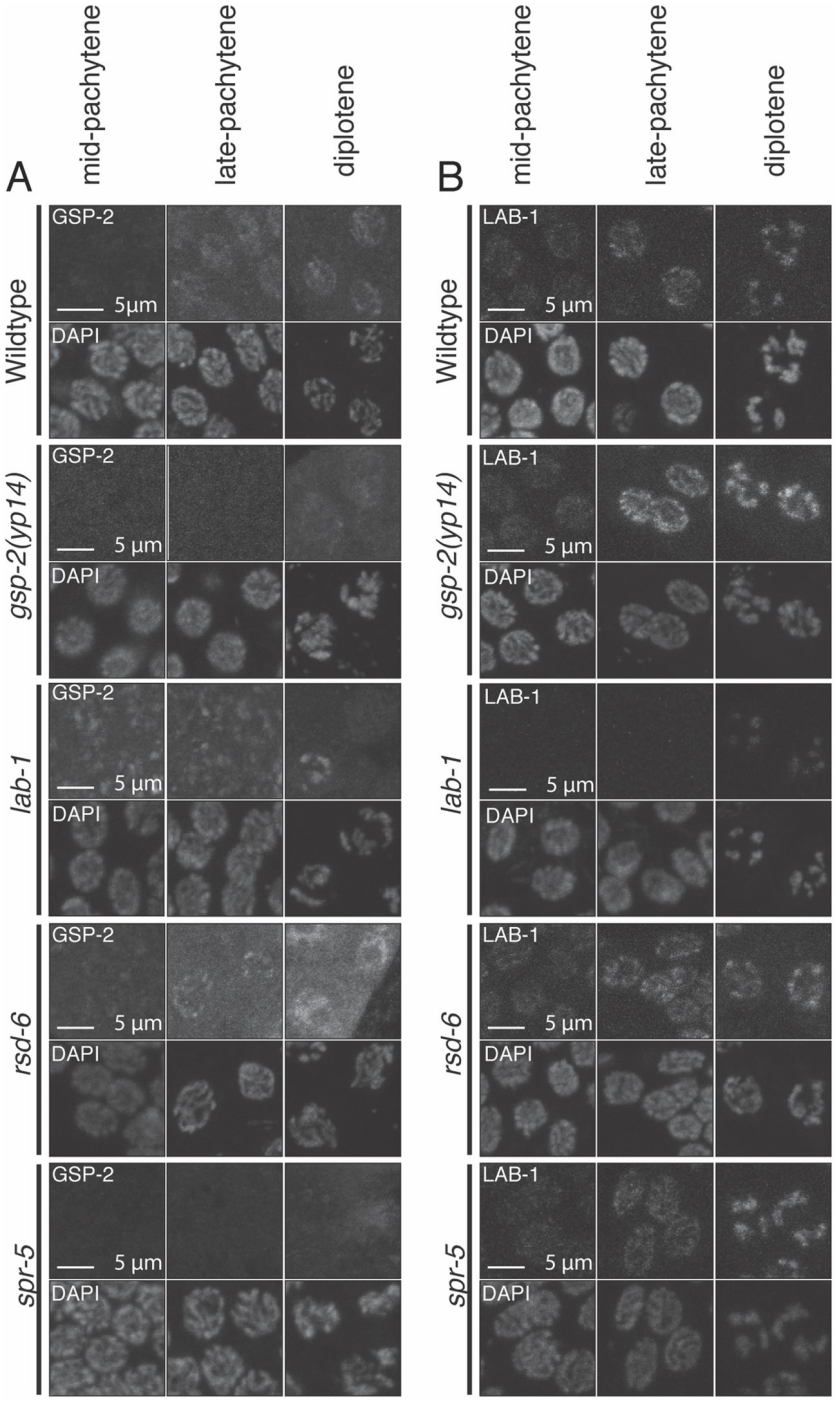
GSP-2 and LAB-1 localization during mid-pachytene to late diplotene. **(A-B)** Representative images of nuclei from early pachytene to late diplotene for controls, *rsd-6*, *gsp-2(yp14)*, *spr-5* and *lab-1* are stained with an antibody against GSP-2 (A) and an antibody against LAB-1 (B). DAPI images for each nucleus are shown to indicate the specific cell cycle stage. All worms were grown at 25°C for 2 generations and fixed as day 2 adults.

To further characterize the nature of the *gsp-2(yp14)* mutation, we examined the localization of LAB-1 and GSP-2 in pachytene nuclei of *gsp-2(yp14)*, *lab-1*, *rsd-6* and *spr-5* animals. Decreased GSP-2 localization was observed in both *gsp-2(yp14)* and *spr-5* mutants but not in *lab-1* or *rsd-6* mutants (Fig 5A). Similar defects in small RNA profiles of *gsp-2* and *spr-5* mutants are consistent with the localization of GSP-2 being normal in *rsd-6* mutants but absent in *gsp-2(yp14)* and *spr-5* mutants (Fig 5A), which supports the possibility that GSP-2 may promote genomic silencing in response to small RNAs. The presence of GSP-2 staining in the *lab-1* deletion was surprising as animals treated with RNAi against *lab-1* show decreased GSP-2 staining. However, as the *tm1791* deletion is a non-null allele, it is possible that GSP-2 can still interact with LAB-1 to some degree. Additionally, we saw no change in LAB-1 localization in any strain except for the *lab-1* deletion, which still exhibited some staining consistent with the *tm1791* deletion being a non-null allele (Fig 5B). Lastly, we assessed LAB-1 localization at diakinesis to determine if LAB-1 localization on the long arms was altered in any of these mutants and we found that localization was relatively normal in *gsp-2(yp14)*, *rsd-6* and *spr-5* mutants (S6 Fig). The localization of LAB-1 in *gsp-2(yp14)* along the long arms was abnormal looking but clearly did not localize to both the long and short arms of the chromosomes.

### *gsp-2(yp14)* and *lab-1* display increased histone H3 phosphorylation

**Fig 6:**
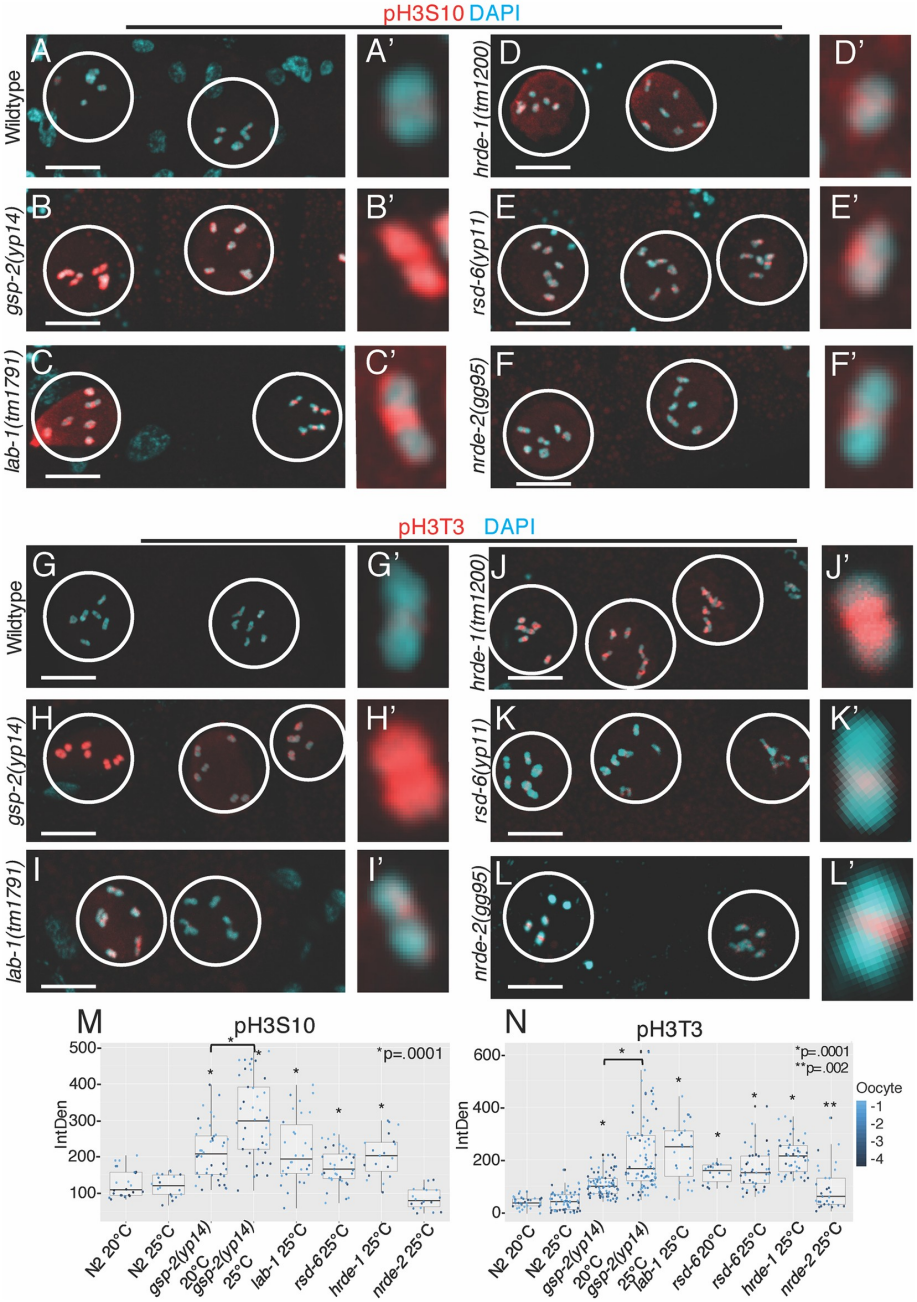
Increased histone phosphorylation is present in *gsp-2(yp14)* oocytes. **(A-F)** Day 2 late stage adults passaged at 25°C stained with an pH3S10 antibody (red) and DAPI marking the DNA (cyan). All samples were prepared at the same time and imaged using identical settings. (A) Wildtype control oocytes show low levels of H3S10p on condensed chromosomes. (B) *gsp-2(yp14)* oocytes have increased levels of H3S10p covering the entire chromosomes. (C,E, F) *lab-1*, *rsd-6* and *hrde-1* mutants also display increased levels of H3S10p but *nrde-2* (D) did not. (N≥20**) (G-L)** Day 2 late generation or sterile adults passaged at 25°C stained with an H3S10p antibody (red) and DAPI marking the DNA (cyan). All samples were prepared at the same time and imaged using identical settings. (G) Control oocytes show low levels of localized H3T3p on the condensed chromosomes. (H) *gsp-2(yp14)* oocytes contain high levels of H3T3p that are expanded to cover the entire condensed chromosome. **(I-L)** *lab-1*, *hrde-1*, *rsd-6* and *nrde-2* all display varying levels of increased H3T3p staining compared to wildtype controls. (N≥20) **(M)** Quantification of fluorescence intensity of H3S10p staining in N2, *gsp-2(yp14)* animals grown at 20°C and 25°C, *rsd-6*, *nrde-2*, *hrde-1*, and *lab-1* shows significant difference in staining intensity between N2 and mutants grown at the same temperature (except for *nrde-2*) and between *gsp-2(yp14)* mutants grown at 20°C and 25°C. (N≥20) **(N)** Quantification of fluorescence intensity of H3T3p staining in N2, *gsp-2(yp14)* animals grown at 20°C and 25°C, *rsd-6*, *nrde-2*, *hrde-1*, and *lab-1* shows significant difference in staining intensity between N2 and mutants grown at the same temperature and between *gsp-2(yp14)* mutants grown at 20°C and 25°C. (N≥20) Scale bar = 10um.

A previously identified phenotype of *gsp-2* null mutants is an increase in Histone 3 Serine 10 (H3S10) phosphorylation due to expansion of the AIR-2-localizing domain [24,30]. In wildtype worms grown at 20°C and 25°C, H3S10 phosphorylation was visible on the condensed chromosomes in the -1 to -3 oocytes, which are defined relative to the spermatheca with the closest being called -1 (Fig 6A, Results summarized S6 Table). In both early- and late-generation *gsp-2(yp14)* mutant oocytes, H3S10 phosphorylation increased when compared with wildtype controls, with increased levels on chromosomes (Fig 6B and 6M). Late-generation *gsp-2(yp14)* mutant animals grown at 25°C had a small but significant increase in H3S10 phosphorylation levels compared to *gsp-2(yp14)* mutant controls grown at 20°C (Fig 6M). Furthermore, we observed increased levels of H3S10 phosphorylation in *lab-1* mutants (Fig 6C and 6M), consistent with previous results [27]. By quantification of fluorescence intensity we measured significant increased levels of H3S10p in *lab-1*, *rsd-6*, and *hrde-1* but not in *nrde-2* mutants (Fig 6C–6F and 6M). The distinct phosphorylation levels in *nrde-2* mutants could reflect its small RNA genome silencing function, where NRDE-2 works downstream of RSD-6 and HRDE-1 to promote accumulation of stalled RNA polymerase II at loci that are targeted by small RNAs [45]. This would suggest that the maintenance of histone marks occurs at the point in the pathway where RSD-6 and HRDE-1 function but not downstream at level of NRDE-2.

PP1 has been previously shown to dephosphorylate a number of histone amino acids, including Histone 3 Threonine 3 (H3T3) [46]. When we examined H3T3 phosphorylation in wildtype controls grown at 25°C, staining was visible in the -1 to -3 oocytes (Fig 4G and 4G’, Results summarized S6 Table). However, in sterile generation *gsp-2(yp14)* mutants, H3T3p staining was significantly brighter than controls when images were taken under the same conditions (Fig 6H and 6H’). Sterile generation *lab-1* and the small RNA mutants *hrde-1*, *rsd-6* and *nrde-2* all exhibited increased H3T3 phosphorylation signal intensity in the -1 to -3 oocytes (Fig 6I, 6L and 6N). Furthermore, there was a significant increase in H3T3 phosphorylation in sterile generation *gsp-2(yp14)* mutant adults compared to the earlier, fertile generation animals suggesting transgenerational accumulation of H3T3 phosphorylation (Fig 6N). Together, our results suggest that an increase in phosphorylation of H3T3 consistently occurs in oocytes of *gsp-2* and small RNA silencing mutants however, increased H3S10 phosphorylation occurs only in *gsp-2(yp14)*, *lab-1*, *rsd-6*, and *hrde-1* but not in *nrde-2* mutants. This defect is sensitive to temperature, as observed for the meiotic chromosome segregation and germ cell immortality defects of *gsp-2(yp14)* (Fig 1E and 1F).

### Methylation of silencing related histone marks is decreased in small RNA genome silencing, *gsp-2* and *lab-1* animals

**Fig 7:**
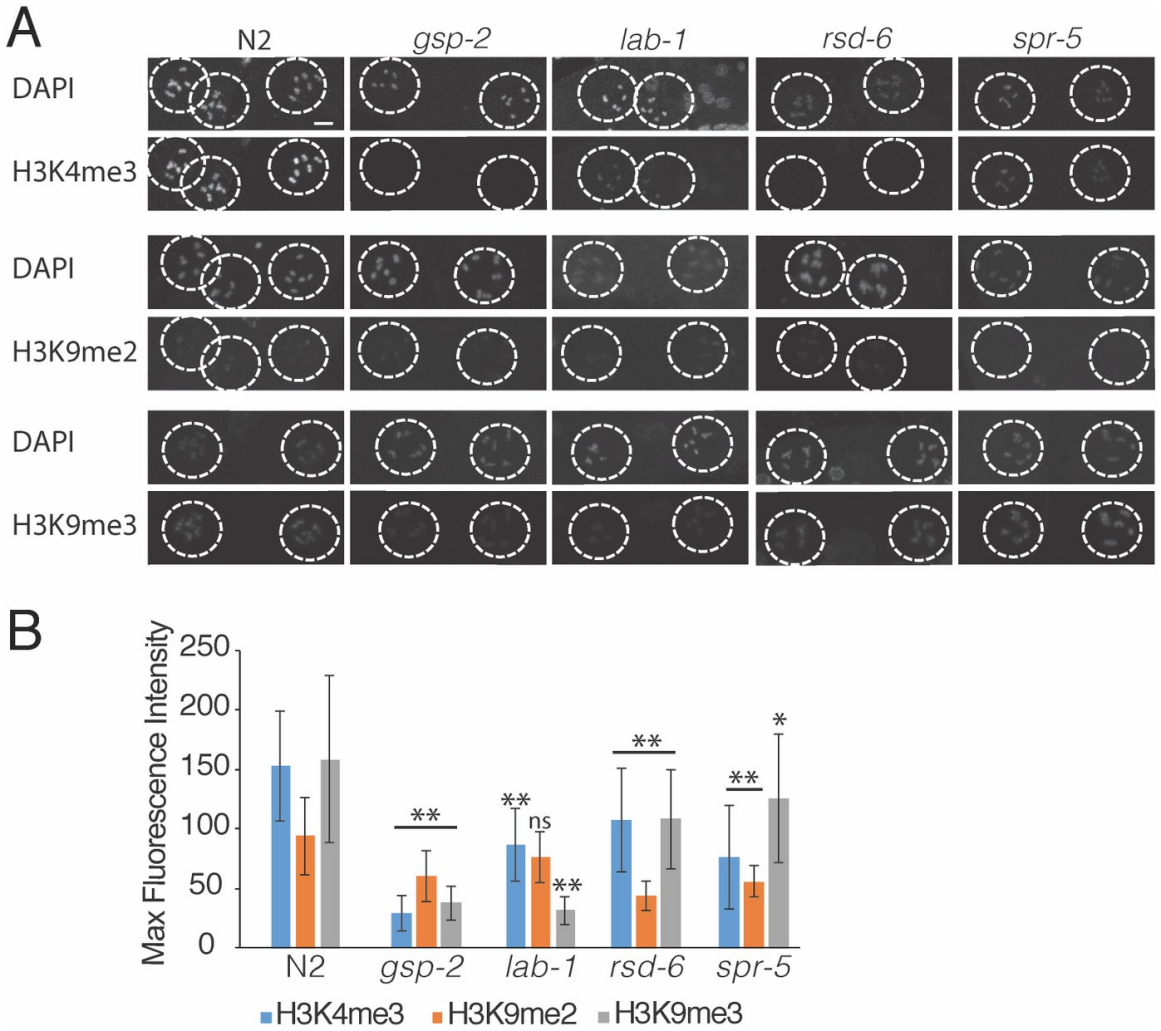
Temperature-sensitive germline immortality exhibit decreased histone methylation. **(A-B)** Control, *rsd-6*, *gsp-2(yp14)*, *spr-5* and *lab-1* animals were grown for 2 generations at 25°C and stained for H3K9me2, H3K9me2 and H3K4me3. (A) Images of diakinesis nuclei are shown (B) Intensity measurements for diakinesis nuclei (N≥41). Error bars for all panels indicate standard deviation.

Finally, we examined histone marks that promote gene silencing or activation. H3K9 methylation can be deposited at silenced genomic loci, and H3K9me and H3S10p marks can function as a phospho-methyl switch where H3S10 phosphorylation can block some epigenetic regulators, such as HP1, from accessing the adjacent H3K9me mark [47–49]. In late-generation fertile *gsp-2(yp14)*, *lab-1*, *rsd-5* and *spr-5* mutant animals grown at 25°C, we observed a significant decrease in H3K9me2 and H3K9me3 intensity in diakinesis oocytes (Fig 7A and 7B, Results summarized S6 Table). We also assessed the H3K4me3 transcriptional activation mark and found that it was significantly decreased in all mutant genotypes at diakinesis (Fig 7A and 7B). It is possible that excess H3T3 phosphorylation present in these mutant strains (Fig 6G–6N) could affect the activities of enzymes that modify histone H3, especially H3K4. Additionally, the presence of excess phosphorylation on adjacent amino acids could perturb the binding of the histone methylation antibodies, possibly disrupting our ability to assess methylation levels.

## Discussion

**Fig 8:**
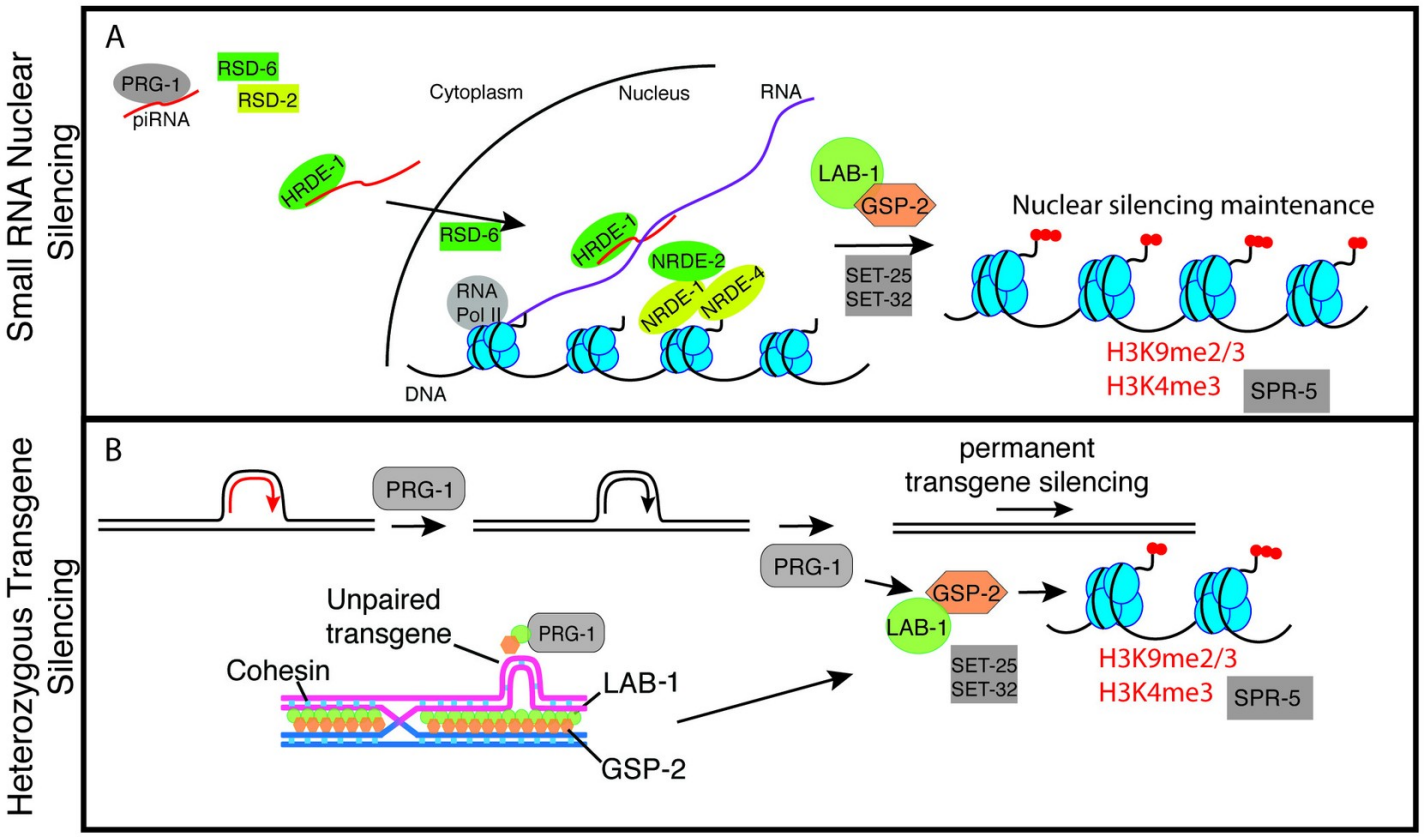
A model for the roles of GSP-2 and small RNA-mediated silencing in promoting germline immortality. We propose that both GSP-2 and small RNA-mediated silencing regulate the transgenerational inheritance of the epigenome. When these pathways are disrupted loss of epigenetic regulation can lead to germline atrophy. **(A**) GSP-2 modulates small RNA silencing machinery promoting small RNA silencing potentially through histone dephosphoryation in a manner that promotes epigenetic silencing, **(B)** Previous work has shown that PRG-1 is important for heterozygous transgene silencing (red = active transgene and black = silenced) in a similar manner to GSP-2. GSP-2/LAB-1 could function to silence small heterozygous regions of DNA, which disrupt meiotic pairing between homologs or cohesion between sister chromatids. This model reflects data presented here and ideas and data from other studies. The model is meant to provoke thoughtful experiments, rather than to represent concepts for which there is definitive experimental proof.

We demonstrate for the first time that *gsp-2* and *lab-1* are required for germ cell immortality at 25°C as strains deficient for these proteins become sterile when they are passaged for several generations (Fig 1C and 1D). Although PP1/GSP-2 is a general protein phosphatase with roles in a number of cellular processes including mitosis and meiosis [33], we identified a separation-of-function allele of *gsp-2* that displayed an X chromosome non-disjunction phenotype that was specific for meiosis (Fig 1B and 1G, S1D and S1E Fig). The incidence of both X chromosome loss and inviable embryos, which are likely aneuploid for autosomes, was exacerbated at high temperature (Fig 1A and 1B), which is consistent with the temperature-sensitive defect in germ cell immortality observed for *gsp-2(yp14)* mutants. Stronger defects in segregation of the X chromosome of *gsp-2(yp14)* mutants during meiosis could be due to the fact that X chromosomes tend to have more central crossovers than the autosomes [50]. PP1/GSP-2 is known to be recruited to meiotic chromosomes by the *C*. *elegans*-specific protein LAB-1, and we found that deficiency for *lab-1* elicited transgenerational sterility accompanied by adult germ cell degeneration phenotypes that were observed in sterile small RNA silencing mutants (Figs 1F and 4). Together, these results indicate that LAB-1 and GSP-2/PP1 are likely to define a critical step during meiosis that potentiates genomic silencing and germ cell immortality (Fig 8). Our data that GSP-2 acts in the context of hemizygous transgenes suggests that it may promote genomic silencing at a stage of germ cell development where homologous chromosomes physically interact.

We found that *gsp-2(yp14)* mutants were proficient for nuclear RNA interference and for the initial generation of silencing of a *GFP* transgene in response to an exogenous dsRNA trigger (Fig 2A and 2B). However, in subsequent (inheriting) generations, *gsp-2(yp14)* mutants failed to maintain GFP transgene silencing, indicating that *gsp-2(yp14)* is defective for RNAi inheritance (Fig 2B), a trait that is frequently associated with temperature-sensitive defects in germ cell immortality [16]. Consistently, propagation of an ‘unpaired’ hemizygous GFP transgene for multiple generations resulted in complete transgene silencing for wildtype controls, but only partial transgene silencing in a *gsp-2(yp14)* mutant background (Fig 2C). These independent tests indicate that *gsp-2(yp14)* is deficient for small RNA-mediated genomic silencing.

Hemizygous transgene silencing occurs in a manner that depends on *prg-1*/Piwi and associated piRNAs as well as downstream factors that promote second siRNA biogenesis [23]. However, we found that piRNA levels were normal in *gsp-2(yp14)* mutants, and also that late-generation *gsp-2(yp14)* strains displayed changes in 22G RNA levels that were similar to those of *spr-5* histone H3K4 demethylase mutants but not to those of *rsd-6* small RNA biogenesis mutants (Fig 2). Moreover, epistasis analysis indicated that there is a weak additive effect when *gsp-2* is combined with the nuclear Argonaute *hrde-1* or the small RNA biogenesis factor *rsd-6*, but no additive effect when *gsp-2* is combined with *nrde-2* (Fig 3) [45]. The parallels with *spr-5* and *nrde-2* mutants suggest that GSP-2 may help to integrate histone silencing modifications with the response to small RNAs (Fig 8). In this context, the GSP-2 phosphatase could directly modify histones or a component of the genome silencing machinery that responds to small RNAs. It is possible that the *yp14* mutation compromises the ability of GSP-2 to interact with either LAB-1 or with small RNA genome silencing proteins in a manner that abrogates the process of small RNA-mediated genomic silencing.

Hemizygous transgenes cause persistent transgenerational discontinuities in the local pairing of small regions of DNA during meiosis, which promotes transgene silencing in a manner that depends on GSP-2 (Figs 2C and 8). Although deficiency for LAB-1 perturbs the pairing of homologous chromosomes during meiosis [26,30], we found that homolog pairing is normal for *gsp-2(yp14)* mutants. LAB-1 localizes to the interface between homologous chromosomes during pachytene, and LAB-1 recruits GSP-2 to nuclei during early stages of meiosis (Fig 8) [26,27,30]. We therefore suggest that LAB-1/GSP-2 may act at the interface between homologous meiotic chromosomes to promote small RNA-mediated epigenomic silencing (Fig 8). An intriguing possibility is that locally ‘unpaired’ hemizygous transgenes could create a structural discontinuity between paired homologous chromosomes that alters the normal meiotic function of LAB-1/GSP-2, creating an environment where the chromosome silencing machinery can respond to small RNAs (Fig 8). Alternatively, the presence of a homologous allele could provide protection from silencing [51–53].

In mammals, a wave of piRNA production occurs during the pachytene stage of meiosis [54,55]. Pachytene piRNAs are derived from intergenic regions, are depleted for transposons, and their functions are not well understood [56]. Given that LAB-1/GSP-2 localizes to the interface between homologous chromosomes during pachytene [26,30], we suggest that one purpose of pachytene piRNAs may be to detect and coordinate the response to ‘unpaired’ structural discontinuities that represent *de novo* transposition events that threaten genome integrity (Fig 8). Consistently, components of the *C*. *elegans* small RNA-mediated genome silencing machinery, such as the HRDE-1 and PRG-1/Piwi Argonaute proteins, are expressed throughout germ cell development and are present during meiotic prophase [6,10,13,18].

Consistent with our results, an allele of the *Drosophila* Protein Phosphatase 1 gene, *Su var (3) 6*, was identified as a suppressor of position-effect variegation, which relieves epigenetic silencing of a transcriptionally active gene that is placed adjacent to a segment of heterochromatin [34]. As position-effect variegation is promoted by small RNA-mediated genome silencing in animals, plants and fungi [57,58], we conclude that PP1 is likely to play a conserved role in this epigenomic silencing process. It has been suggested that the heterochromatin defect of *Su var (3) 6* mutants could reflect a direct role of PP1 in dephosphorylation of H3S10p, a mark that results in dissociation of Heterochromatin Protein 1 from heterochromatin [46,59]. Moreover, human PP1 has been shown to dephosphorylate H3T3p, this function is also carried out by *C*. *elegans* GSP-2 during meiosis [46,59]. One or both of these silencing marks could be relevant to meiotic small RNA-mediated genome silencing.

We propose that the role of LAB-1/GSP-2 in genome silencing may be at a stage of meiosis, possibly pachytene, when LAB-1/GSP-2 are localized between paired homologs in a manner that might be capable of responding to small ‘unpaired’ discontinuities between homologs like hemizygous transgenes (Fig 8). This model raises questions about the significance of increased H3T3 and H3S10 phosphorylation levels in mature oocytes of *gsp-2(yp14)* mutants at diakinesis when homologous chromosomes are held together only by chiasma [26,27]. H3 phosphorylation defects were not observed at earlier stages of germ cell development, but similarly increased levels of H3T3 phosphorylation were observed at diakinesis for small RNA genome silencing mutants (Fig 7A and 7B). This could suggest that altered histone phosphorylation levels could be an indirect effect of dysregulation of heterochromatin, which could affect the activity of a protein that functions in the context of heterochromatin, such as the H3T3 kinase Haspin [36]. It is also possible that the diakinesis-specific phosphorylation defect that we observed reflects a fundamental property of how GSP-2 promotes genomic silencing in response to small RNAs. For example, the structure that triggers genomic silencing could occur at pachytene when homologous chromosomes are paired, but the role of GSP-2 in responding to small RNAs could occur at a later stage of germ cell development like diakinesis, potentially via H3 phosphorylation.

Work in several organisms, particularly in fungi and *Drosophila*, has shown that local regions of heterozygosity are prone to silencing during meiosis in a small RNA dependent manner (reviewed by [22]). Our study defines a meiotic process that links transgenerational small RNA-mediated genome silencing with the structure of paired homologous chromosomes during meiosis. Given that endogenous small RNAs promote germ line stem cell maintenance, oogenesis and meiosis itself [60,61], we suggest that small RNA pathways and germ cell development have evolved to become mutually reinforcing processes.

## Materials and methods

### Strains

All strains were cultured at 20°C or 25°C on Nematode Growth Medium (NGM) plates seeded with *E*. *coli* OP50. Strains used include Bristol N2 wild type, *gsp-2(tm301) III*, *gsp-2(yp14) III*, *lab-1(tm1791) I*, *cpIs12[Pmex-5*::*GFP*::*tbb-2 3'UTR + unc-119(+)] II*, *hrde-1(tm1200) III*, *rsd-6(yp11) I*, *nrde-2(gg95) II*, *rbr-2(tm1231) IV*, *smc-3(t2553) III*, *coh-4(tm1857) V*, *coh-3(gk112) V*, *air-2(or207) I*, *unc-32(e189) III*, *unc-13(e450) I*, *unc-24(e1172) IV*. *smc-3(t2553)* is a temperature sensitive missense mutation, and *coh-4(tm1857)/coh-3(gk112)* are deletions.

### Germline mortality assay

Worms were assessed for the Mortal Germline phenotype using the assay previously described [2]. L1 or L2 larvae were transferred for all assays. After passaging plates that yielded no additional L1 animals were marked as sterile. Log-rank analysis was used to determine differences of transgenerational lifespan between strains.

### DAPI staining

DAPI staining was performed as previously described. L4 larvae were selected from sibling plates and sterile adults were singled as late L4s, observed 24 hours later for confirmed sterility, and then stained 48 hours after collection.

### RNA FISH

DNA oligonucleotide probes coupled with a 5′ Cy5 fluorophore were used to detect repetitive element expression. The four probes used in this study were as follows: tttctgaaggcagtaattct, *CeRep59* on chromosome *I* (located at 4281435–4294595 nt); agaattactgccttcagaaa, antisense *CeRep59* on chromosome *I*; caactgaatccagctcctca, chromosome *V* tandem repeat (located at 8699155–8702766 nt); and gcttaagttcagcgggtaat, 26S rRNA. The strains used for RNA FISH experiments were *rsd-6(yp11)*, *gsp-2(yp14)*, and N2 Bristol wild type. Staining was performed as described by Sakaguchi et al., 2014.

### Immunofluorescence

Adult hermaphrodites raised at 20°C or 25°C were dissected in M9 buffer and flash frozen on dry ice before fixation for 1 min in methanol at -20°C. After washing in PBS supplemented with 0.1% Tween-20 (PBST), primary antibody diluted in in PBST was used to immunostain overnight at 4 °C in a humid chamber. Primaries used were 1:500 pH3S10 (Millipore, 06570), 1:4000 pH3T3 (Cell Signaling, D5G1I, Rabbit) 1:50 GSP-2 antibody (Colaiacovo lab), 1:300 LAB-1 antibody (Colaiacovo lab), 1:200 HIM-8 antibody raised in guinea pig (Dernburg lab), 1:200 SYP-1 antibody raised in goat, 1:500 H3K9me3 (Abcam ab8898), 1:500 H3K9me2 (Milipore Upstate 07–441), 1:500 H3K4me3 (Active Motif 39159). Secondary antibody staining was performed by using an Cy3 donkey anti-mouse or Cy-5 donkey anti-rabbit overnight at 4°C. All images were obtained using a LSM 710 laser scanning confocal and were taken using same settings as control samples. Images processed using ImageJ and Icy (http://icy.bioimageanalysis.org/). Intensity quantification was done by measuring total fluorescence in individual condensed chromosomes and subtracting the background levels obtained from mitotic nuclei as nucleoplasm levels varied greatly. Histone methylation intensity measurements were measured without background subtraction since only very few background was present.

### RNAi assays

N2 wildtype, *gsp-2*, *rsd-6* and *nrde-2* animals were grown on *lin-26* RNAi clones and the progeny of 10 worms each were scored for Embryonic Lethality.

### Transgene silencing assay

*cpIs12* and *gsp-2; cpIs12* worms were scored for GFP expression on NGM plates and then transferred to RNAi plates targeting GFP. The next generation (that was laid on GFPi plates) were scored for GFP expression and their sisters were removed and transferred back to NGM plates. Worms were propagated for multiple generations on NGM and scored each time for GFP expression. Both GFP reporter *gsp-2* doubles were created by marking with *dpy-17*.

### Heterozygous transgene silencing

*cpIs12* was maintained as a heterozygote over *dpy-10 unc-4* for *gsp-2* strains that were heterozygous for *cpIs12*, *gsp-2* remained mutant for the entire assay and *cpIs12* was balanced over *dpy-10 unc-4*.

### Genome sequence analysis

Paired sequence reads (2X100 nucleotide long) were mapped to the C. elegans reference genome version WS230 (www.wormbase.org) using the short-read aligner BWA [62]. The resulting alignment files were sorted and indexed with the help of the SAMtools toolbox [63]. The average sequencing depths for the mutant and wild-type N2 strains were 116x and 71x, respectively. Single-nucleotide variants (SNVs) were identified and filtered with SAMtools and annotated with a custom Perl script using gene information downloaded from WormBase WS230. Candidate SNVs in the mutant strain already present in the N2 strain were eliminated from further consideration.

The raw sequence data from this study have been submitted to the NCBI BioProject (http://www.ncbi.nlm.nih.gov/bioproject) under accession number PRJNA395732 and can be accessed from the Sequence Read Archive (SRA; https://www.ncbi.nlm.nih.gov/sra) with accession number SRP113543.

### Small RNA sequence analysis

5’ independent small RNA sequencing was performed as described previously [13], using one repeat for each time-point of N2 wildtype, *rsd-6* and *spr-5* at 25°C. Custom Perl scripts were used to select different small RNA species from the library. To map small RNA sequences to genes, reads were aligned to the *C*. *elegans* ce6 genome using Bowtie, Version 0.12.7, requiring perfect matches [64]. Data was normalized to the total number of aligned reads and 1 was added to the number of reads mapping to each gene to avoid division by zero errors. To map 22G sequences to transposons and tandem repeats, direct alignment to the transposon consensus sequences, downloaded from Repbase (Ver 17.05) or repeats obtained from the ce6 genome (WS190) annotations downloaded from UCSC as above, was performed using Bowtie allowing up to two mismatches and reporting only the best match. Uncollapsed fasta files were used for these alignments to compensate for the problem of multiple identical matches. Data was normalized to the total library size and 1 was added to the number of reads mapping to each feature to avoid division by zero errors. In order to analyze data from *rsd-2* mutants grown at 20°C [65], Fasta files were downloaded from the Gene Expression Omnibus and uncollapsed using a custom Perl script before aligning to transposons or tandem repeats as above. Analysis of data was carried out using the R statistical language [66].

The small RNA sequencing data from this study are available from GEO database accession number GSE126531.

## Supporting information

S1 Fig Mapping and non-complementation test of *gsp-2(yp14)*. **(A)** Map of genomic region surrounding *gsp-2* on Chr. *III*. **(B)** Mapping of *gsp-2(yp14)* between *dpy-17* and *unc-32* on Chr. *III* placing *yp14* at -1.08. **(C)** Non-complementation test for Him phenotype between *gsp-2(yp14)* and *gsp-2(tm301)* showed an incidence of males of 5.7% at 20°C. **(D-E)** Analysis of incidence of males showed no jackpots of males at in *gsp-2(yp14)* animals when shifted as L1’s to 25°C or as L4’s to 25°C. **(F)** F1 *gsp-2(yp14)/+* progeny scored for HIM do not exhibit a HIM phenotype.
(TIF)

S2 Fig Repetitive regions in the genome are desilenced in *gsp-2(yp14)* animals. **(A-F)** Confocal images of Cy5-labeled RNA FISH probes (green) and DAPI-stained nuclei (blue). **(A,C,E)** RNA FISH probes show expression of Ch V repeats in the germlines of *gsp-2(yp14)* (C) and *rsd-6* (E) animals grown at 25°C and only embryonic expression in wildtype controls (A). **(B,D,F)** Probes against CeRep59 repeats reveal similar germline expression in *gsp-2(yp14)* (D) and *rsd-6* (F) animals and embryo-only expression in wildtype controls (B). All images were taken under the same condition. The germ line is outlined with white line. Scale bar = 30um.
(TIF)

S3 Fig *spr-5* and *gsp-2* show overlap in their small RNA populations. **(A)** Multigenerational inheritance assay using a second transgene *pkls32* in the background of *hrde-1* and *gsp-2* mutants. **(B-E)** Comparison of small RNAs in *rsd-6*, *gsp-2* and *spr-5* mutants grown at 25°C: **(B)** *rsd-6* vs *gsp-2*, **(C)** *spr-5* vs *gsp-2*, **(D)** *rsd-6* vs *spr-5* and **(E)** N2 vs *gsp-2*. **(F)** Global 22G-RNA levels relative to the levels of small RNAs in early generation N2 wildtype grown at 25°C, for the indicated strain grown at 25°C. Boxplots show interquartile range, with a line at the median and with whiskers extending to the furthest point that is < = 1.5 times the interquartile range from the median. **(G)** miRNA levels relative to miRNAs in early generation N2 wildtype grown at 25°C, for the indicated strain grown at 25°C. Interquartile range and whiskers are as for (F).
(TIF)

S4 Fig *lab-1* but not *gsp-2(yp14)* exhibited significant pairing defects in late pachytene. **(A)** Images show HIM-8 localization at mid-pachytene for control, *rsd-6*, *gsp-2(yp14)*, *spr-5* and *lab-1* animals. **(B)** To quantify pairing each germline was divided in 5 equal zones illustrated here.
(TIF)

S5 Fig Loss of cohesion or *gsp-2(tm301)* did not cause germline atrophy. **(A)** 100% of adult *gsp-2(tm301)* animals displayed normal germline size by DAPI staining (N = 30). P-values present in S4 and S5 Tables. Scale bar = 10um. **(B)** DAPI staining and germline analysis showed no germline atrophy in *smc-3* and *coh-3; coh-4* mutants and minor defects in *air-2* animals suggesting loss of chromosome cohesion alone does not cause germline atrophy. (N = 30).
(TIF)

S6 Fig LAB-1 localized normally at diakinesis in *gsp-2(yp14)* and small RNA genome silencing mutants. Images show LAB-1 and SYP-1 localization at early to late diakinesis for control, *rsd-6*, *gsp-2(yp14)*, *spr-5* and *lab-1*. One chromosome was magnified to show proper localization on the long and short chromosome arms.
(TIF)

S1 Table Expected vs observed embryonic lethality. (DOCX)

S2 Table Wilcox test comparing log2(normalized 22G LATE+1)-log2(normalized 22G EARLY +1) to log2 (normalized 22G LATE N2+1)-log2(normalized 22G EARLY N2+1). (DOCX)

S3 Table Wilcox test comparing normalized miRNAs to normalized miRNAs in N2 early [alternative = less]. (DOCX)

S4 Table P-values for adult germline defects in *gsp-2* and temperature-sensitive small RNA mutants. (DOCX)

S5 Table P-values for L4 germline defects in *gsp-2* and temperature-sensitive small RNA mutants. (DOCX)

S6 Table Summary of results. (DOCX)
