# Supplementary figures and images for: The meiotic phosphatase GSP-2/PP1 promotes germline immortality and small RNA-mediated genome silencing
Source: PLoS Genet. 2019 Mar 28;15(3):e1008004. doi: 10.1371/journal.pgen.1008004 (PMC6456222; doi:10.1371/journal.pgen.1008004)

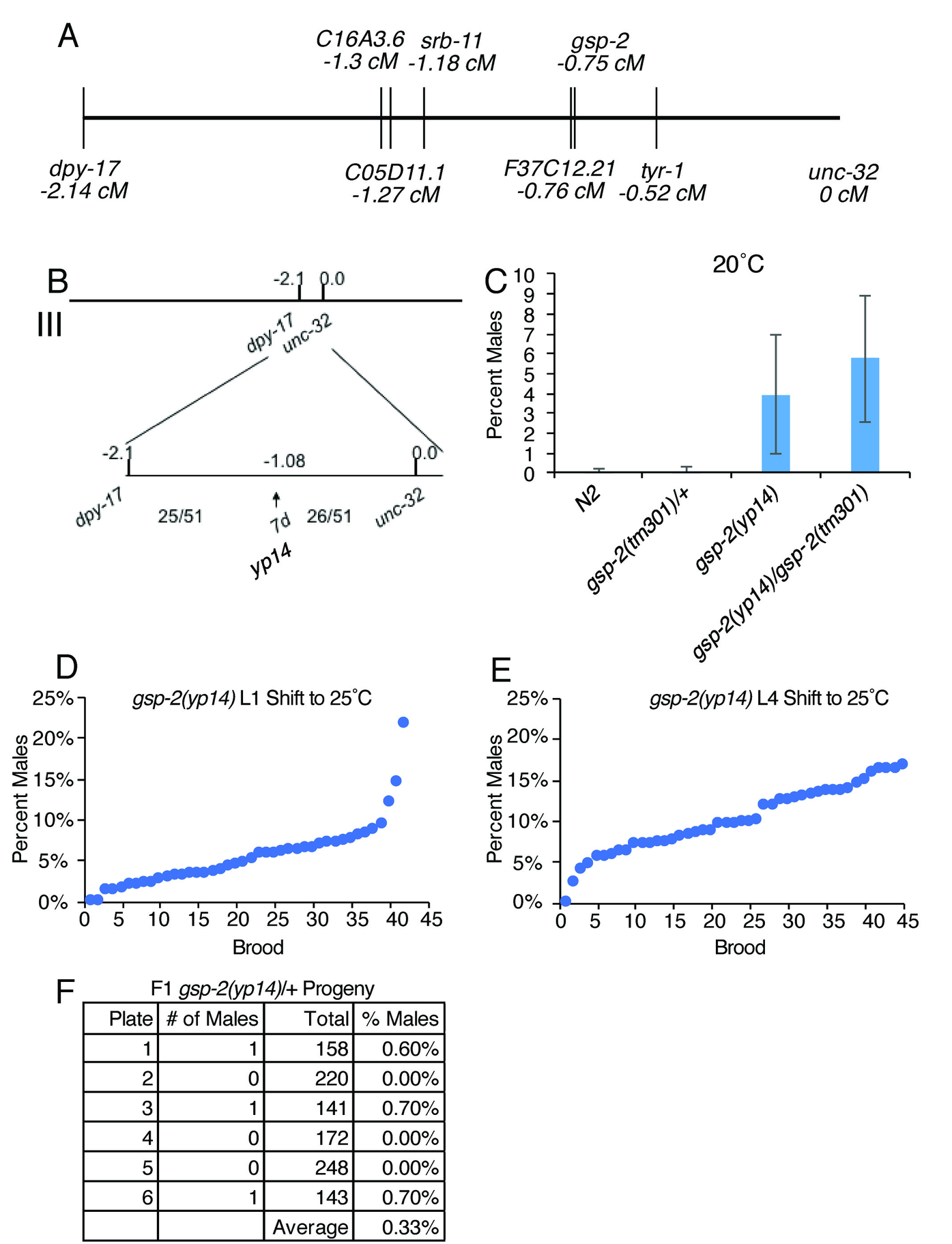

Supplement: S1 Fig — (A) Map of genomic region surrounding gsp-2 on Chr. III. (B) Mapping of gsp-2(yp14) between dpy-17 and unc-32 on Chr. III placing yp14 at -1.08. (C) Non-complementation test for Him phenotype between gsp-2(yp14) and gsp-2(tm301) showed an incidence of males of 5.7% at 20°C. (D-E) Analysis of incidence of males showed no jackpots of males at in gsp-2(yp14) animals when shifted as L1’s to 25°C or as L4’s to 25°C. (F) F1 gsp-2(yp14)/+ progeny scored for HIM do not exhibit a HIM phenotype. (TIF) [file pgen.1008004.s001.tif]

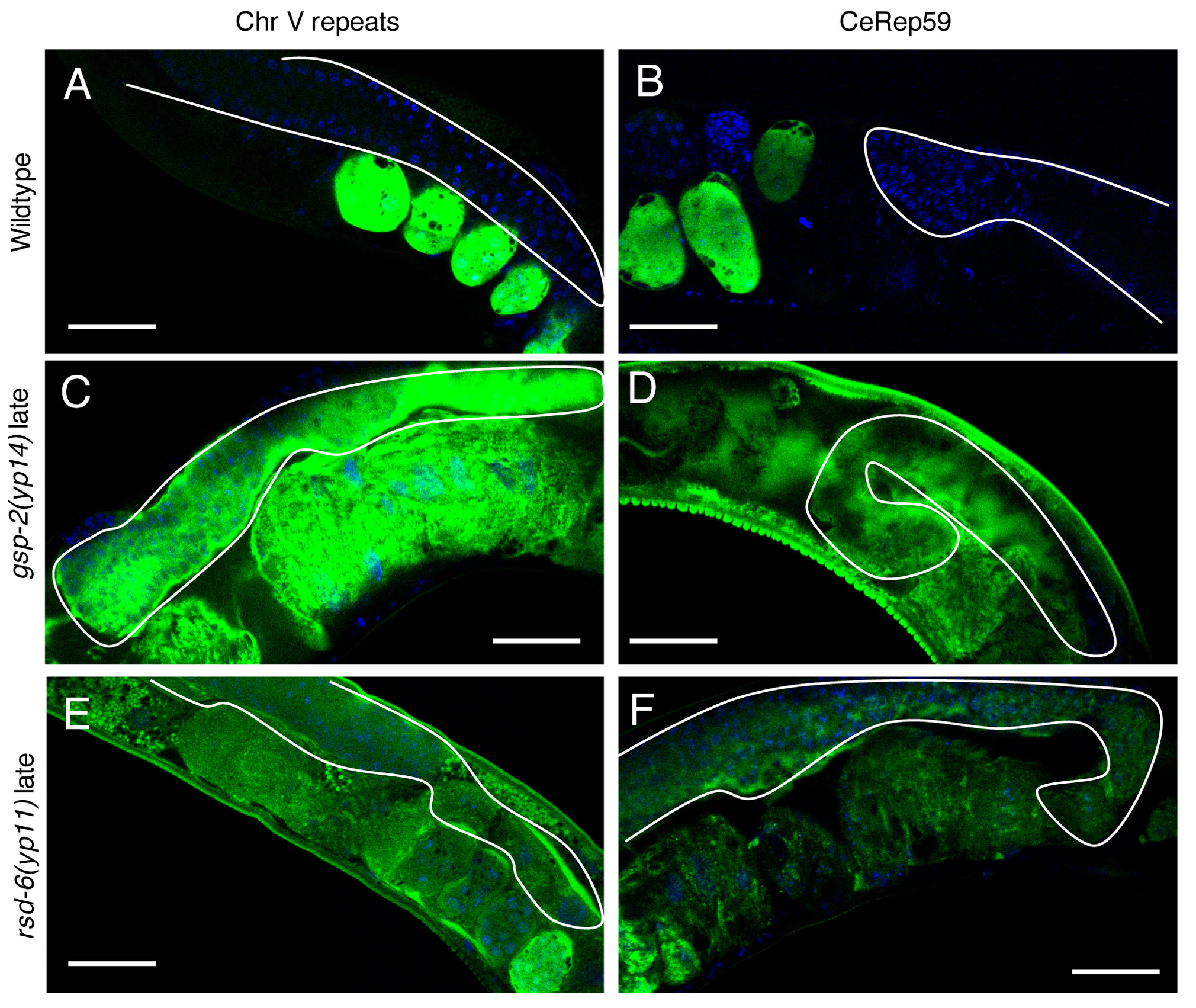

Supplement: S2 Fig — (A-F) Confocal images of Cy5-labeled RNA FISH probes (green) and DAPI-stained nuclei (blue). (A,C,E) RNA FISH probes show expression of Ch V repeats in the germlines of gsp-2(yp14) (C) and rsd-6 (E) animals grown at 25°C and only embryonic expression in wildtype controls (A). (B,D,F) Probes against CeRep59 repeats reveal similar germline expression in gsp-2(yp14) (D) and rsd-6 (F) animals and embryo-only expression in wildtype controls (B). All images were taken under the same condition. The germ line is outlined with white line. Scale bar = 30um. (TIF) [file pgen.1008004.s002.tif]

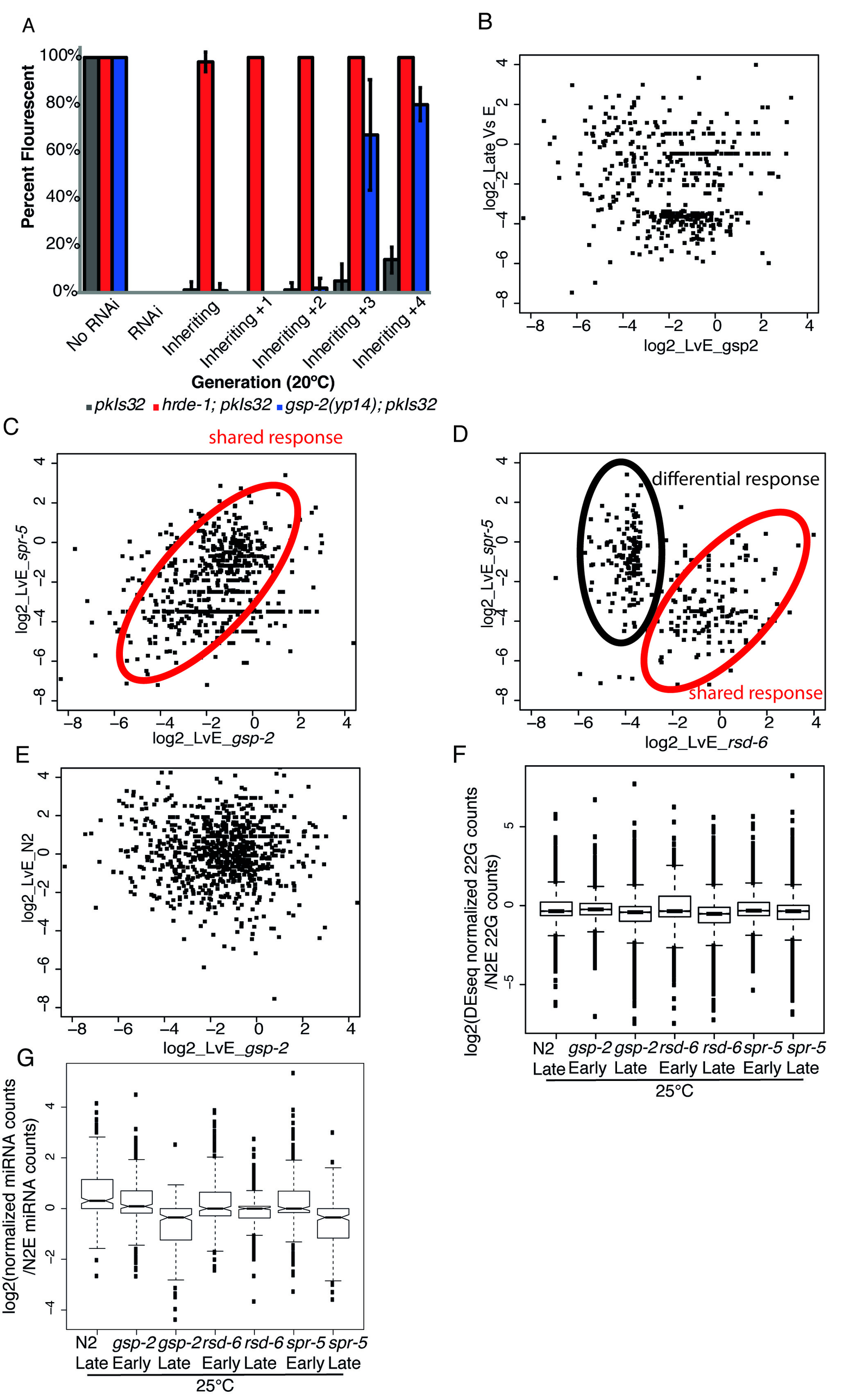

Supplement: S3 Fig — (A) Multigenerational inheritance assay using a second transgene pkls32 in the background of hrde-1 and gsp-2 mutants. (B-E) Comparison of small RNAs in rsd-6, gsp-2 and spr-5 mutants grown at 25°C: (B) rsd-6 vs gsp-2, (C) spr-5 vs gsp-2, (D) rsd-6 vs spr-5 and (E) N2 vs gsp-2. (F) Global 22G-RNA levels relative to the levels of small RNAs in early generation N2 wildtype grown at 25°C, for the indicated strain grown at 25°C. Boxplots show interquartile range, with a line at the median and with whiskers extending to the furthest point that is < = 1.5 times the interquartile range from the median. (G) miRNA levels relative to miRNAs in early generation N2 wildtype grown at 25°C, for the indicated strain grown at 25°C. Interquartile range and whiskers are as for (F). (TIF) [file pgen.1008004.s003.tif]

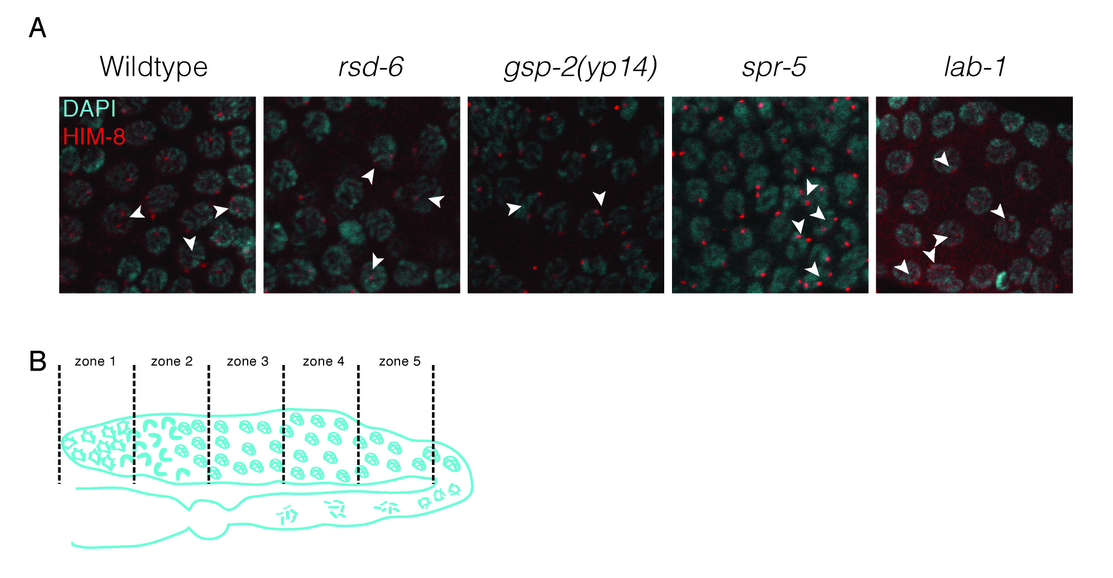

Supplement: S4 Fig — (A) Images show HIM-8 localization at mid-pachytene for control, rsd-6, gsp-2(yp14), spr-5 and lab-1 animals. (B) To quantify pairing each germline was divided in 5 equal zones illustrated here. (TIF) [file pgen.1008004.s004.tif]

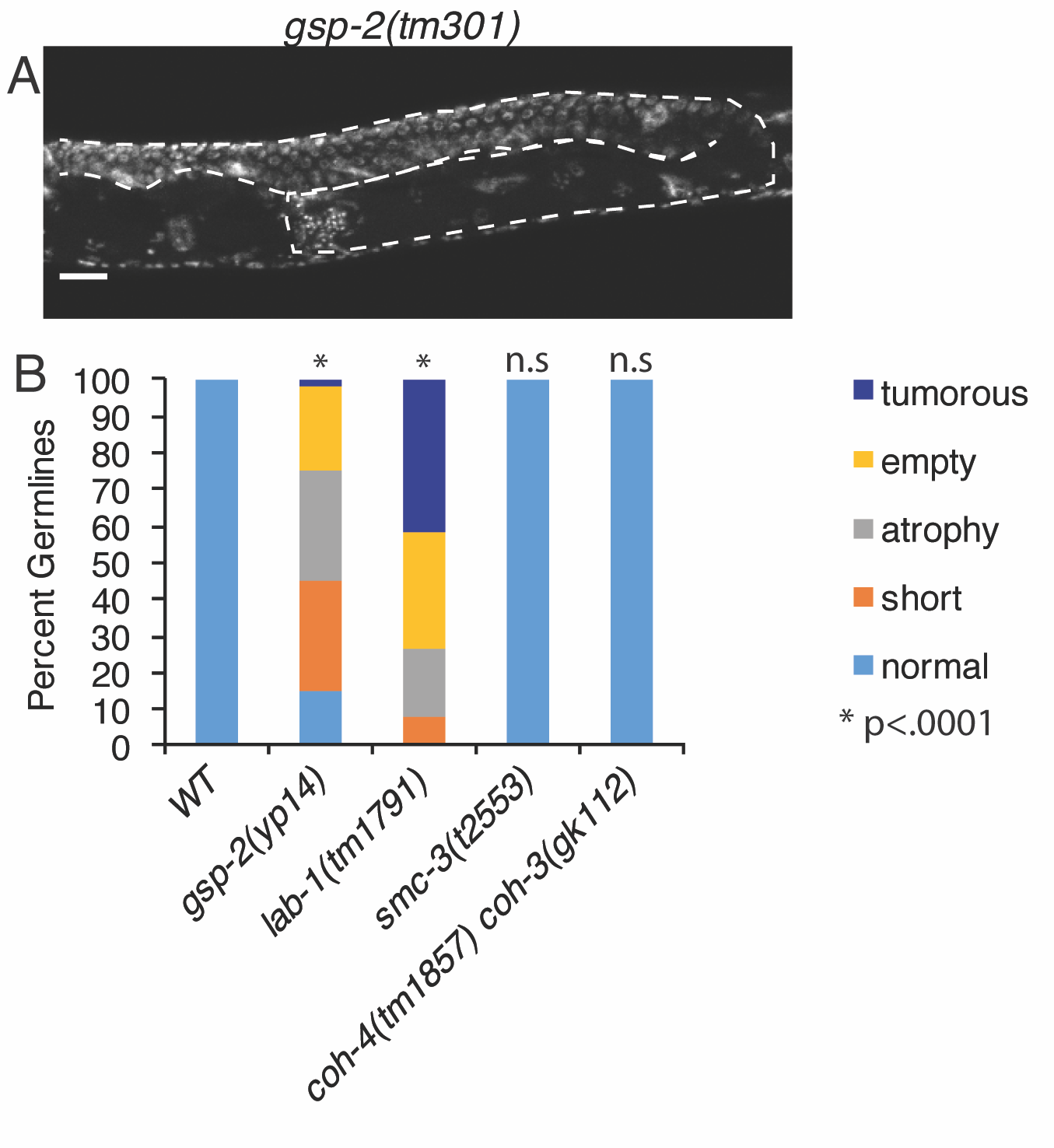

Supplement: S5 Fig — (A) 100% of adult gsp-2(tm301) animals displayed normal germline size by DAPI staining (N = 30). P-values present in S4 and S5 Tables. Scale bar = 10um. (B) DAPI staining and germline analysis showed no germline atrophy in smc-3 and coh-3; coh-4 mutants and minor defects in air-2 animals suggesting loss of chromosome cohesion alone does not cause germline atrophy. (N = 30). (TIF) [file pgen.1008004.s005.tif]

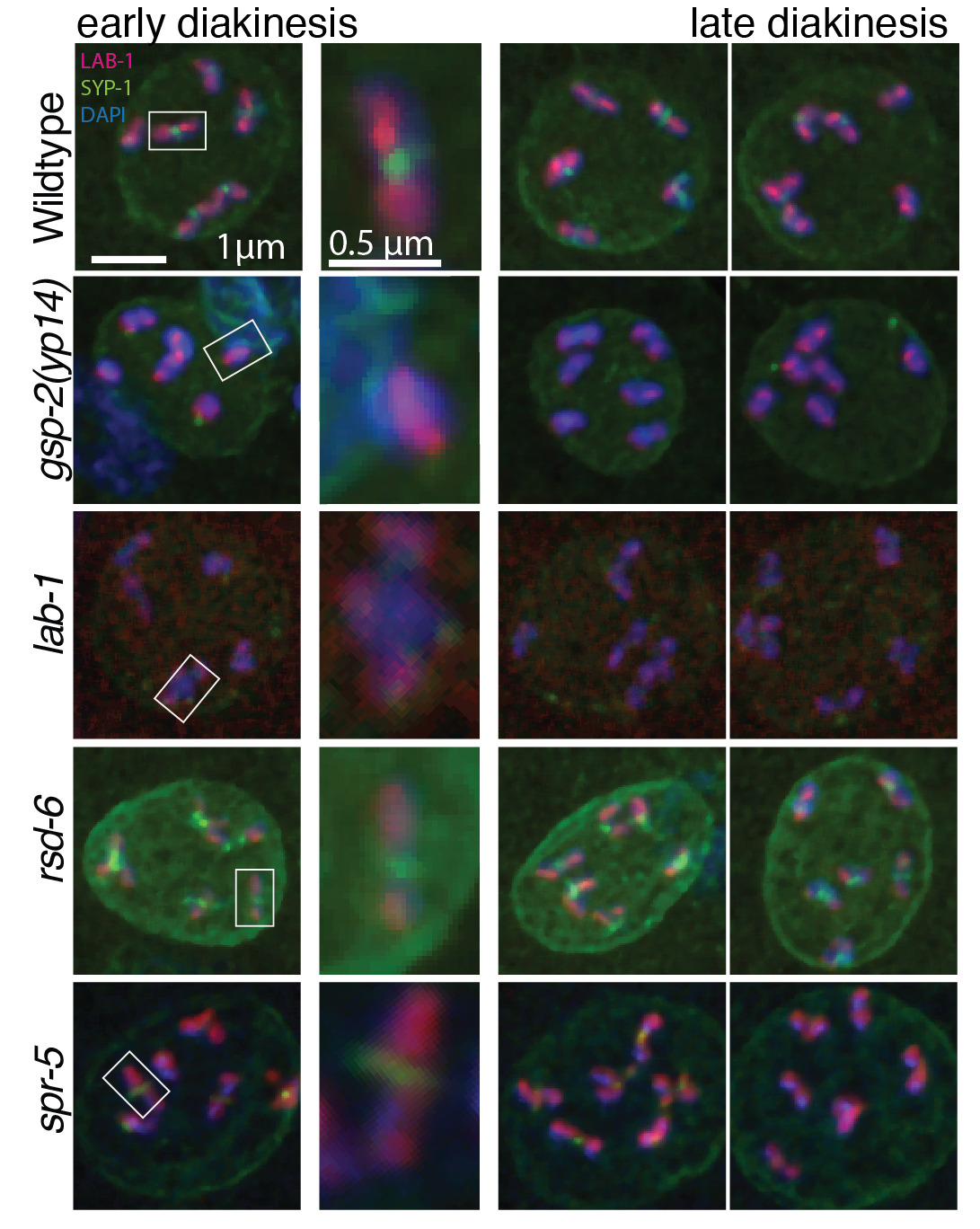

Supplement: S6 Fig — Images show LAB-1 and SYP-1 localization at early to late diakinesis for control, rsd-6, gsp-2(yp14), spr-5 and lab-1. One chromosome was magnified to show proper localization on the long and short chromosome arms. (TIF) [file pgen.1008004.s006.tif]
