# Supplementary material for: The meiotic phosphatase GSP-2/PP1 promotes germline immortality and small RNA-mediated genome silencing
Source: PLoS Genet. 2019 Mar 28;15(3):e1008004. doi: 10.1371/journal.pgen.1008004 (PMC6456222; doi:10.1371/journal.pgen.1008004)
Supplement: S1 Table — (DOCX) [file pgen.1008004.s007.docx]

|  | **Males** | **Exp Dead Emb** | **Exp Live Worms** | **Obs Dead Emb** | **Obs Live Worms** | **P-value** |
| --- | --- | --- | --- | --- | --- | --- |
| ***20°C gsp-2(yp14)*** | 164 | 820 | 5290 | 88 | 1492 | <0.0001 |
| ***25°C gsp-2(yp14)*** | 155 | 775 | 964 | 481 | 1380 | <0.0001 |

**Supplemental Table 1: Expected vs Observed Embryonic Lethality**
