# Supplementary material for: The meiotic phosphatase GSP-2/PP1 promotes germline immortality and small RNA-mediated genome silencing
Source: PLoS Genet. 2019 Mar 28;15(3):e1008004. doi: 10.1371/journal.pgen.1008004 (PMC6456222; doi:10.1371/journal.pgen.1008004)
Supplement: S2 Table — (DOCX) [file pgen.1008004.s008.docx]

**Supplemental Table 2: Wilcox test comparing log2(normalized 22G LATE+1)-log2(normalized 22G EARLY +1) to log2( normalized 22G LATE N2+1)-log2(normalized 22G EARLY N2+1)**
