# Supplementary material for: The meiotic phosphatase GSP-2/PP1 promotes germline immortality and small RNA-mediated genome silencing
Source: PLoS Genet. 2019 Mar 28;15(3):e1008004. doi: 10.1371/journal.pgen.1008004 (PMC6456222; doi:10.1371/journal.pgen.1008004)
Supplement: S4 Table — (DOCX) [file pgen.1008004.s010.docx]

**Supplemental Table 4: P-values for adult germline defects in *gsp-2* and temperature-sensitive small RNA mutants**
